# Magnetic guidewire steering at ultrahigh magnetic fields

**DOI:** 10.1126/sciadv.adg6438

**Published:** 2023-04-26

**Authors:** Mehmet Efe Tiryaki, Yiğit Günsür Elmacıoğlu, Metin Sitti

**Affiliations:** ^1^ Physical Intelligence Department, Max Planck Institute for Intelligent Systems, 70569 Stuttgart, Germany.; ^2^ Institute for Biomedical Engineering ETH, Zurich, 8092 Zurich, Switzerland.; ^3^ School of Medicine and College of Engineering, Koç University, 34450 Istanbul, Turkey.

## Abstract

With remote magnetic steering capabilities, magnetically actuated guidewires have proven their potential in minimally invasive medical procedures. Existing magnetic steering strategies, however, have been limited to low magnetic fields, which prevents the integration into medical systems operating at ultrahigh fields (UHF), such as magnetic resonance imaging (MRI) scanners. Here, we present magnetic guidewire design and steering strategies by elucidating the magnetic actuation principles of permanent magnets at UHF. By modeling the uniaxial magnetization behavior of permanent magnets, we outline the magnetic torque and force and demonstrate unique magnetic actuation opportunities at UHF, such as in situ remagnetization. Last, we illustrate the proposed steering principles using a magnetic guidewire composed of neodymium magnets and a fiber optic rod in a 7-Tesla preclinical MRI scanner. The developed UHF magnetic actuation framework would enable next-generation magnetic robots to operate inside MRI scanners.

## Teaser

Ultrahigh field magnetic actuation enables magnetic guidewire steering inside MRI scanners.

## INTRODUCTION

Despite over a decade-long development of magnetic resonance imaging (MRI) intervention techniques and tools, MRI still fights an ever-losing battle in endovascular interventions against x-ray fluoroscopy (*1*–*3*). The radiologists praise the ionizing radiation-free nature and superior soft tissue contrast of MRI; however, the high imaging rate, operational comfort, and easy accessibility of x-ray fluoroscopy make interventionalist prefer x-ray fluoroscopy over MRI. Even the improved image feedback rate of real-time MRI sequences (*4*–*6*) and the developments in MRI-compatible intervention tools could not tip the scale toward interventional MRI (*7*). The major challenges of current interventional MRI systems are the limited and noncomfortable workspace inside the MRI scanner and much lower MRI resolution due to the reduced MRI main static field. Therefore, in the last decade, researchers have proposed various robotic actuation methods for interventional MRI to eliminate such drawbacks (*1*, *8*).

These actuation methods are divided into two main categories: externally powered actuation methods, such as tendon-driven (*9*, *10*), hydraulic (*11*, *12*), concentric tube (*13*, *14*), and shape memory alloy (SMA)-based actuation (*15*, *16*); and MRI-powered magnetic methods, such as Lorentz force (*17*–*22*), and magnetic force–based actuation (*23*–*26*). Externally powered interventional tools provide precise control, smooth manipulation, and minimal interference to the MR images; however, they suffer from mechanical nonlinearities, limited access to torturous anatomy, and downscaling problems due to complex internal structures, such as tendons and pressurized tubes (*1*). Moreover, they have complex driving mechanisms that further obstruct the limited workspace in the MRI scanner (*9*–*14*). SMA-based actuation reduces the complexity by applying local forces (*15*, *16*); however, SMA results in temperature-rise concerns due to actuator heating and RF coupling with the MRI scanner. On the other hand, MRI-powered actuation methods lead to more compact designs, benefiting remote magnetic torque and force applied at the distal tip of the guidewire for steering. For instance, the Lorentz force–based active catheters use the high axial field of MRI for torque generation to create considerable bending at the tip of the catheters. Not requiring a complex mechanical driving mechanism, these active catheters are a promising option for MRI interventions. Studies even showed that the MRI-guided Lorentz force–based catheters might have comparable performance with standard x-ray fluoroscopy (*19*, *20*). However, Lorentz force–actuated catheters require high driving currents causing heating problems similar to SMA-based actuators (*18*) and a substantial amount of current-passing wiring near the MRI, creating health risks for the interventionalist and the patient. Moreover, the wirings in the catheter shaft can create unpredictable tension problems during the navigation in tortuous structures and radio frequency (RF) coil-induced heating problems during MRI.

A completely remote MRI-powered actuation approach uses a ferromagnet or a permanent magnet at the distal tip of the guidewire, which results in an intervention tool as simple as precurved guidewires. The magnetic guidewire can then be steered in three-dimensional (3D) space using magnetic pulling forces generated by MRI’s gradient coil hardware (*23*–*26*). Since magnetic guidewires do not require electrical current and have a relatively small conductive material, they do not cause any heating problems. While magnetic gradient–based pulling offers an intuitive actuation mechanism and safe design in terms of heating, it has two fundamental problems for widespread use. First, it requires real-time software access to the MRI scanner’s gradient coil hardware (*26*). However, most commercial MRI systems do not provide direct access to such real-time software. Second, the MRI gradient coils can provide low-amplitude magnetic gradients; therefore, a large magnetic guidewire tip (*25*) or an additional high-power gradient coil setup is needed in the MRI scanner (*23*, *24*). An alternative magnetic actuation method is using the MRI scanner as a magnetic force trap that magnetically pulls in the distal magnetic tip of the guidewire while the magnet is constrained by guidewire tension and vascular structure. Azizi *et al.* (*27*) proposed fringe field navigation (FFN) by demonstrating that a small permanent magnet at the distal tip of the guidewire could pull the guidewire into the distal blood vessels using the fringe fields outside of an MRI scanner. However, steering a guidewire using outside MRI fringe fields requires a large industrial robotic arm manipulating the patient in 3D space near the MRI scanner. Such 3D patient manipulation around the MRI scanner could be impractical for the clinical scenarios since the physicians should continuously adjust their position relative to the patient rotation. Moreover, the previously proposed fringe fields–based guidewire navigation concept only benefits the magnetic forces, while the steering strategies could be expanded beyond magnetic pulling by considering magnetic actuation holistically at the ultrahigh field (UHF).

**Fig. 1.**
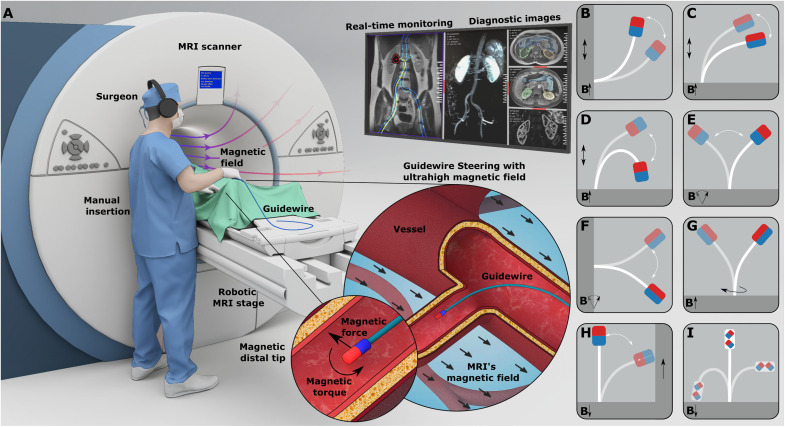
Magnetic guidewire actuation concept at UHF inside an MRI scanner. (**A**) A clinical scenario of UHF actuation. The surgeon manually steers the guidewire using the magnetic fields inside the MRI scanner by observing real-time feedback from MRI images. (**B** to **D**) Guidewire bending by insertion to the MRI scanner for different magnetization configurations. The guidewire with three different configurations is presented: parallel (B), perpendicular (C), and antiparallel (D) configurations. The guidewire at a different stage of motion is overlaid. The MRI scanner’s magnetic field direction is shown with an arrow. The insertion direction is shown with double arrows. (**E** and **F**) Guidewire bending by a rotation platform for parallel and perpendicular configurations. The rotating magnetic field direction shows the platform rotation. The parallel configuration rotates the guidewire tip around the MRI’s magnetic field direction. The perpendicular configuration rotates the guidewire perpendicular to the MRI’s magnetic field. The guidewire at a different stage of motion is overlaid. (**G**) Base twist actuation of the guidewire with a perpendicular magnet. The base twist is shown with a rotational arrow. The guidewire at a different stage of motion is overlaid. (**H**) In situ magnetization switching from antiparallel to parallel configuration during an insertion inside a constrained space. The guidewire is initially in the antiparallel configuration and leans over the solid wall during insertion into the MRI scanner due to the magnetic torque. Later, the magnetization direction switches and aligns with the MRI field. (**I**) The dual-magnet guidewire in parallel, perpendicular, and antiparallel configurations. The magnetization direction is shown for each separate configuration.

Further elaborating the continuous magnetization model of uniaxial permanent magnets and magnetic actuation principles at the high fields (*28*–*31*), in this study, we present a canvas of UHF magnetic guidewire steering strategies in the MRI scanner (Fig. 1). By challenging the common assumption that the magnetic objects are fully aligned with the ultrahigh MRI main field (*8*, *27*), we observed that the uniaxial permanent magnets operating at saturation magnetization could hold magnetization nonparallel to the MRI main field and magnet’s easy axis. Then, we designed magnetic guidewires comprising a fiber optic rod as an elastic core and a magnetic element at the distal end (Fig. 1A). The oblique magnetization vector combined with the elastic guidewire body allowed us to achieve different bendings by balancing the magnetic and elastic torques and forces by placing the patient in different positions relative to the MRI’s main field. We proposed different magnetic easy axis configurations of the tip magnet, including parallel (Fig. 1B), perpendicular (Fig. 1C), and antiparallel (Fig. 1D) configurations, which could be used for steering at different insertion angles. Also, we demonstrated that motions of the patient, such as the patient’s insertion into an MRI scanner (Fig. 1, B to D) and rotation relative to the main field direction (Fig. 1, E and F), could be used for orienting the distal tip, and the base twist of the guidewire with a perpendicular magnet could also be used as an independent steering mode similar to precurved guidewires without moving the patient. Moreover, we also showed unique magnetic actuation opportunities arising at UHF, such as magnetization direction switching during navigation. In situ magnetization direction switch could be used during navigation for the transition from antiparallel to parallel configuration and vice versa (Fig. 1H) and dual-magnet configuration to switch the overall stable axis of the guidewire during navigation (Fig. 1I). Last, we demonstrated magnetic steering capabilities in physiologically relevant 3D vasculature phantoms with the arterial flow and while MRI in a porcine kidney ex vivo.

## RESULTS

### Magnetization of uniaxial permanent magnets at UHF

**Fig. 2.**
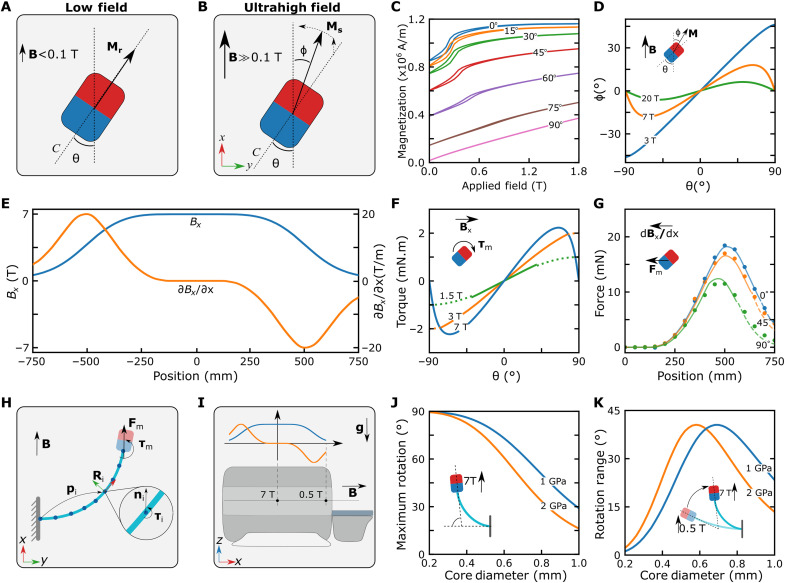
Magnetic actuation method at UHF. (**A**) Magnetization at low fields, *B* < 0.1 T. The red and blue colors represent the direction of the easy axis (*C*). (**B**) Magnetization at high fields, *B* >> 0.1 T. The permanent magnet is magnetized substantially by the external magnetic field. (**C**) Magnetization curves of the cylindrical neodymium magnet measured inside a vibrating sample magnetometer at different θ. The magnet’s magnetization in the *x* direction is measured, while the magnetic field is swept from 0 to 1.8 T in the *x* direction. (**D**) Magnetization vector alignment as a function of the easy axis alignment at different external field strengths. (**E**) Magnetic fields and gradients in the *x* direction of the MRI scanner. (**F**) Model-based magnetic torque acting on the cylindrical magnet as a function of the magnet orientation. The magnet is in its saturation regime for the solid lines and nonsaturated regime for the dashed lines. The direction of the torque is shown on the schematic. (**G**) Magnetic force as a function of the magnet position in the MRI scanner for different magnet orientations. The solid lines represent the force estimated with the magnetization model using the MRI magnetic field and gradient measurements. The dashed lines are the linear model estimation in the nonsaturated region. The dots are the force sensor measurements in the experiments. (**H**) The discretized Cosserat rod model, including the magnetic actuation. (**I**) Schematic of the magnetic actuation simulations. (**J** and **K**) The maximum rotation and rotation range in the simulations as a function of the guidewire thickness.

Permanent magnets, such as neodymium magnets, are the most commonly used magnetic materials in magnetic actuation due to their high remanence magnetization allowing high magnetic torque and force transmission at low magnetic fields (*32*–*38*). At low magnetic fields, permanent magnets are modeled with a constant magnetization vector aligned to the magnet’s easy axis (*C*), as shown in Fig. 2A. While constant magnetization assumption leads to minimal error at magnetic fields lower than 100 mT (*30*), it fails to capture physical interaction of magnets with the magnetic field at UHF. Although the magnetization theory of permanent magnetic materials at UHF is well studied in magnetism (*31*), it has not been investigated as a magnetic actuation method in robotics until now due to the limited field strength of commonly used magnetic/electromagnetic actuation systems (*30*).

At UHF, permanent magnets behave similarly to soft magnets (*28*), such that the magnetization vector of the permanent magnet is substantially rotated by the external field (Fig. 2B). Therefore, to calculate the magnetic force and torque acting on the permanent magnets accurately, we need to know the direction of the magnetization vector. In soft magnets, shape anisotropy is the main factor determining the direction of the magnetization at high fields, and the magnetization vector could be calculated by minimizing the internal magnetic energy of the magnet (*28*, *29*). In contrast, crystal anisotropy is the dominant factor determining the magnetization direction of permanent magnets at high fields. While there are many different crystal anisotropy types at the macroscopic scale (*31*), the crystal anisotropy of bulk permanent magnets could be modeled using a uniaxial permanent magnet model. The anisotropy energy of the uniaxial magnets (*E*_a_) is defined as

$$E_{a}=K_{1}\sin^{2}(\phi -\theta )$$ (1)

where *K*_1_ is the anisotropy constant, θ is the angle between the easy axis and the magnetic field, and ϕ is the angle between the magnetization vector and the magnetic field. The direction of the magnetization vector is then calculated by optimizing the following magnetic energy (*E*) term

$$E=\underset{\phi }{\min}K_{1}\sin^{2}(\phi -\theta )-M_{s}H\cos(\phi )$$ (2)

where *H* is the magnitude of the external magnetic field, and *M_s_* is the saturation magnetization of the material. The optimization problem can be solved using open-source nonlinear optimization methods for any θ. However, we first need to measure the magnetic material constants *M*_s_ and *K*_1_.

In this study, we focused on bulk neodymium magnets due to their high saturation magnetization and high anisotropy constant. We estimated the magnetic material constants using a vibrating sample magnetometer (VSM) by measuring the magnetization (*M*_x_) in the external field direction at different angles in Fig. 2C. We used a 1-mm-diameter and 1-mm-height cylindrical neodymium magnet for such measurement. *M*_s_ could be directly observed from the saturation value of measurement at θ = 0° as 1.16 × 10^6^ Am. To estimate the anisotropy constant, *K*_1_, we looked into rotated measurements at their saturation. Because of the limited magnetic field (1.8 T) of the VSM system, we could not saturate the sample at higher angles; therefore, we used saturation magnetization measurements at θ = 15° and 30° to calculate *K*_1_. First, we classified the sample as saturated if *M*_y_^2^ + *M*_x_^2^ > *M*_s_^2^, where *M*_y_ *= M*_r_sin(θ) and *M*_r_ is the remanence magnetization (fig. S1). Next, we predicted the magnetization angle of the saturated measurements using a cosine approximation ϕ_m_ = arccos(*M*_x_/*M*_s_). Then, we calculated *K*_1_ by minimizing (ϕ* − ϕ_m_)^2^ for each data point, where ϕ* is the solution of Eq. 2. We found *K*_1_ = 2.26 ± 0.16 × 10^12^ Am (2.76 ± 0.20 × 10^7^ erg/cm^3^) for the (1-mm diameter and 1-mm height) cylindrical magnet, where ±SD with 95% confidence interval. We also compared the VSM measurements with a (1.8-mm diameter) spherical neodymium magnet to observe the effect of the shape anisotropy (fig. S2). For the spherical magnet, we calculated *K*_1_ = 2.10 ± 0.17 × 10^12^ Am, which showed a minimal effect of the shape on the saturation behavior, as expected (*39*). Then, the nonlinear relation between θ and ϕ was calculated by optimizing the energy term for different saturating field values in Fig. 2D. It can be immediately seen that the magnetization direction converged to the field direction, i.e., ϕ= 0°, as the magnetic field increased, and ϕ had a maximum value depending on the magnetic field. This maximum value can be analytically calculated as

$$\phi_{\max}=\arcsin(K_{1}/M_{s}H)$$ (3)

by setting the derivative of the cost function (energy term) in Eq. 2 to zero, and the corresponding easy axis angle is θ_max_= ϕ_max_ + π/4.

Although we operated at UHF, at which the permanent magnet was saturated, we also investigated the nonsaturation region of the magnetization curve to provide a smooth transition from low to high fields. We observed the nonlinear magnetization behavior and hysteresis effects dominant in the easy axis direction and near linear magnetization behavior close to θ = 90°. We approximated the magnetization at the low field with a linear model,

$$M_{x}(H,\theta )=\chi (\theta )H+M_{r}\cos(\theta )\;\mathrm{and}\;M_{y}=M_{r}\sin(\theta )$$ (4)

where χ is the effective susceptibility, and *H* is the external magnetic field. We calculated χ for θ = 0° using the slope between remanence and saturation point, χ(θ = 0°) *=* (*M*_s_ − *M*_r_)/*H*_s_, where *H*_s_ is the field at which the sample was saturated and for θ = 90° using the slope between 0 and 1.8 T. We calculated χ(θ = 0°) *=* 0.358 and χ(θ = 90°) *=* 0.241. Since angles between 0° and 90° have a slope between these two values, we used the linear interpolation for the slopes in between, χ(θ) = χ_0°_ + θ × (χ_90° −_ χ_0°_)/90. While the linear model for low field resulted in a substantial maximum error up to 0.115 × 10^6^ Am at 400 mT and a mean error of 0.057 ± 0.042 × 10^6^ Am for θ = 0° due to the nonlinearities, it is more important to capture the high angles accurately at low field strengths. Since the sample reached saturation at 1.05 T at θ = 0°, we operated mainly in the saturation regime. Whereas at θ = 90°, the saturation occurred much later, around 4 T, according to our linear model, which covers a substantially larger magnetic field range. At θ = 90°, our linear model captured the magnetization with a relatively small error of 0.015 ± 0.005 × 10^6^ Am between 0 and 1.8 T.

Last, we investigated the effect of hysteresis, which is prominent during magnetization in the easy axis direction. We performed measurements of the magnetization process at θ = 0° to verify the robustness of magnetization with hysteresis. First, we measured the sample’s magnetization while ramping up the applied field, starting from different initial magnetizations (fig. S3A) while ramping down (fig. S3B). As a result, we observed that the magnetization followed parallel curves inside the borders of the slim hysteresis region in Fig. 2C and converged to the same saturation curve, proving that hysteresis did not affect the magnetization at the saturation regime.

### Magnetic actuation in the MRI scanner

To model the magnetic torque and force in the MRI scanner, we measured the magnetic field and magnetic gradient in the axial main field direction (i.e., *x* direction) of the MRI scanner, as shown in Fig. 2E. Using the field measurement, we calculated the magnetization angle ϕ, we wrote the magnetization vector as **M** = *M*_s_ [cos(ϕ), sin(ϕ), 0] in the magnet coordinate frame whose *x* direction is aligned with the main field of the scanner and *x*-*y* plane contains the easy axis of the magnet (Fig. 2B). Then, we can calculate the magnetic torque acting on the permanent magnet using **τ***_m_* = μ_0_*V*(**M** × **H**), and the magnitude of the torque can be calculated as

$$\tau_{m}=\mu_{0}VM_{s}H\sin(\phi )$$ (5)

where μ_0_ is the permeability of the free space, and *V* is the volume of the magnetic object. Calculating the magnetic torque for different magnetic fields (Fig. 2F), we could see that the magnetic torque still increased with the increasing magnetic field at a small θ. However, as θ increased, we observed a nonlinear relation between the torque and magnetic field. First, relatively low fields, such as 1.5 and 3 T, cannot saturate the magnet at high θ. Therefore, we used our linear magnetization model for the nonsaturated region, which assumed constant *M*_y_ until saturation; hence completed the angle torque relation with a sine-like profile and caused only a small discontinuity (Fig. 2F). As the field strength increased further above 4 T, the magnet was saturated completely at all angles. The magnetic torque started decreasing at θ close to 90° and became zero at θ *=* 90° for field strength above 4.6 T. This behavior results in two equilibrium points for UHF: at θ *=* 0° and θ *=* 90°, which are stable and unstable, respectively. While the transition to saturation at exact θ *=* 90° is still unclear, the torque model captures the uniaxial behavior. Furthermore, another interesting observation was that the magnetic torque has a theoretical maximum value τ_max_ = μ_0_*VK*_1_, like the maximum theoretical torque reported for shape anisotropy (*28*). However, crystal anisotropy of the neodymium magnet could withstand larger magnetic field strength than shape anisotropic soft magnets (fig. S4) with the same volume, which is necessary to generate high magnetic torque at UHF (see note S1 for further details). Moreover, the permanent magnet’s ability to provide magnetic torque at an aspect ratio close to 1 increases flexibility in designing guidewires with different magnetization directions.

Because of the fringe fields–based huge magnetic gradients, up to 20 T/m, in the MRI scanner’s bore entrance(Fig. 2E), we must also consider the magnetic force acting on the permanent magnet, which can be calculated by **F**_m_ = μ_0_*V*(**M**·**∇**)**H**, where **∇** is the vector differential operator. In the magnetic force model, we focused on the force in the *x* direction

$$F_{m}=\mu_{0}V\frac{\partial H_{x}}{\partial x}M_{x}$$ (6)

where ∂*H_x_*/**∂***x* is the magnetic gradients in the MRI’s main field axis (*x* direction). To verify the magnetic pulling force acting on the magnet in the axial direction, we attached 1-mm-diameter and 1-mm-height neodymium cylindrical magnets on an MRI-conditioned force sensor at three different angles and measured the force acting in the *x* direction. We observed notable agreement between the experimentally measured force and calculated force from our magnetization model considering the gradients in the *x* direction in Fig. 2G, except mismatch for θ *=* 90° at high fields. The uniaxial model predicted that the *M*_x_ be larger at θ *=* 90° than θ *=* 45°; however, the force measurement was larger at θ *=* 45°, suggesting that the saturation magnetization *M*_s_ was less at θ *=* 90°. Since the VSM could not measure the magnetization at UHF, we fitted *M*_s_ for the magnetic force at θ *=* 90°. We calculated *M*_s_ = 1.02 × 10^6^ Am, which resulted in the model in Fig. 2G with a small discontinuity. Although further understanding the magnetization behavior at high angles is important in terms of comprehensive UHF magnetization modeling, it requires VSM systems with UHF measurement capability. Fortunately, it is possible to achieve large guidewire bendings using different magnet configurations while operating the tip magnet at small magnetization angles in MRI; therefore, this mismatch has minimal effect on the actuation model. Moreover, the magnetic gradients in radial directions also exist in the MRI scanner (fig. S5). However, ∂*H_y_*/∂*y* resulted in much less force, especially at small θ since *M*_y_ is much smaller than *M*_x_ due to the low ϕ at the UHF (fig. S6). Therefore, we neglected the radial forces and only considered the magnetic forces in the axial direction of the MRI scanner in the bending analysis.

### Guidewire design

After understanding the magnetic torque and force acting on the permanent magnet at UHF, we looked into the design of the accompanying flexible body, an MRI-compatible glass fiber optic rod, as the guidewire’s elastic core. The general design principle for magnetically actuated guidewires is optimizing the stiffness of the flexible body according to the steering capacity of the magnetic actuation system (*32*–*34*). However, since the MRI scanner cannot provide a high degree of freedom (DoF) actuation as other external permanent magnet-based actuation systems (*32*–*34*) and electromagnetic actuation systems (*35*–*37*), we cannot limit our design objective only to maximizing the bending. Therefore, we optimized the flexible body stiffness considering two main criteria: maximizing the bending angle at the center of the MRI scanner and the bending angle range as the guidewire is inserted through the bore of the MRI.

We developed a discretized Cosserat rod model–based dynamic simulation environment simulating guidewire shape, including elastic and gravitational forces for the MRI magnetic force and torque trap–based actuation system, to investigate the effect of the elastic core parameters according to these two criteria. In our simulations, we calculated the magnetic torque and forces acting on the magnet by calculating the magnetization direction of the tip magnet through Eq. 2 at each time step by considering the position of the tip magnet in the MRI scanner. Then, the magnetic torque and force at the tip of the guidewire were added to the Cosserat rod model as a constraint to the boundary value problem (Fig. 2H).

We performed bending simulations using a 2-cm-long fix-end guidewire with a parallel tip magnet to choose the fiber thickness. First, a 7-T magnetic field was applied to the guidewire at a 90° initial angle in simulation to calculate the maximum tip bending. Second, the bending range was calculated by simulating the motion of the guidewire from the MRI bore entrance 0.5 T to the center 7 T (Fig. 2I). Then, we repeated the simulations for different effective Young’s moduli and guidewire diameters. We observed that as the guidewire got much softer, the maximum bending at 7 T converged to 90°; however, the bending angle at 0.5 T also converged to 90°; hence the rotation range decreased to 0° (Fig. 2, J and K). Conversely, as the rotation range increased, the maximum bending decreased for the stiffer guidewire. Therefore, we chose a 500-μm-diameter fiber optic with a 1.13 ± 0.01 GPa effective Young’s modulus that compromised the two competing criteria. We used the magnetic guidewires made with this fiber in the rest of the study.

### Magnetic steering modes at UHF

**Fig. 3.**
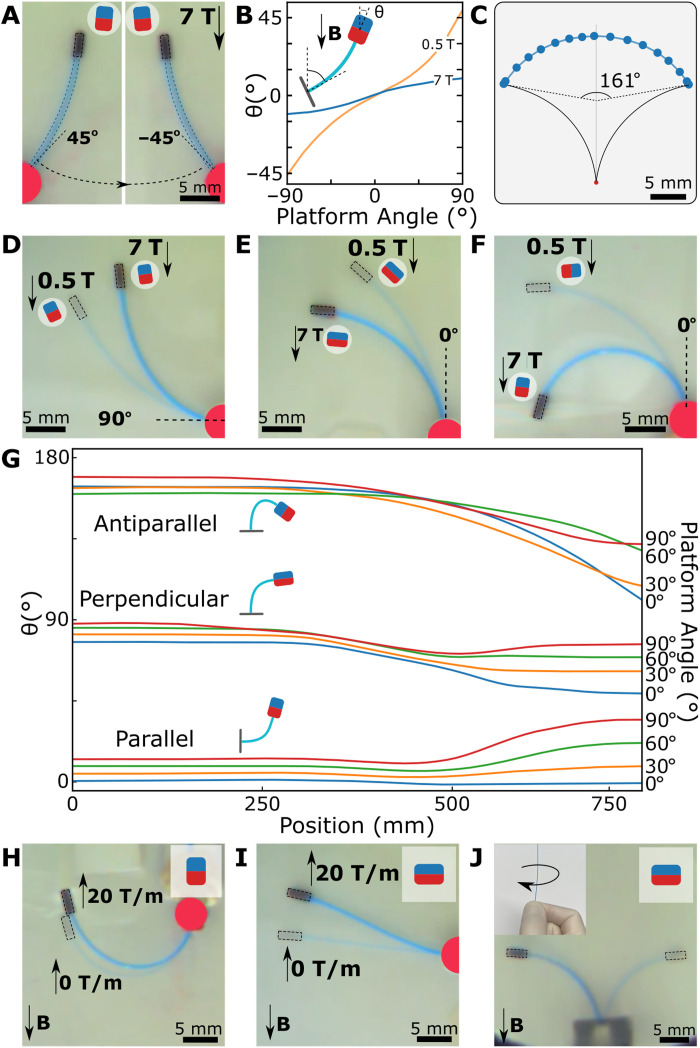
Magnetic steering modes at UHF. (**A**) Experimental image of the guidewire in the parallel magnet configuration at two different platform angles at 7 T. The magnet schematic shows the magnet direction. (**B**) The guidewire tip orientation for different platform angles at 0.5 and 7 T. (**C**) The guidewire workspace in guidewire coordinate frames at 7 T. The rotation limit of the tip is measured as 161°. (**D** to **F**) Experimental images of the guidewire during platform motion in the MRI scanner for parallel (D), perpendicular (E), and antiparallel (F) configurations. The image of the guidewire at 0.5 T is overlaid on the guidewire image at 7 T. (**G**) The guidewire tip orientations during the linear motion platform in the MRI scanner. The platform orientation for each line is given on the right *y* axis as a legend. (**H**) Assistive magnetic gradient pulling at the antiparallel configuration. The experimental image of the guidewire at zero magnetic gradients is overlaid on the image at the maximum magnetic gradient. (**I**) Magnetic gradient for steering the guidewire in the perpendicular configuration. The experimental image of the guidewire at zero magnetic gradients is overlaid on the image at the maximum magnetic gradient. (**J**) Base twist actuation of the guidewire with the perpendicular magnet at 7 T. The guidewire direction is twisted by hand. The images of the guidewire before and after the twist are overlaid.

Magnetic guidewire steering at low fields was achieved through the interaction of high DoF magnetic actuation systems, such as electromagnetic coils (*35*–*37*) and magnetic end-effectors of robotic arms (*32*–*34*). However, in the absence of a high DoF magnetic actuation system, the magnetic configurations of the guidewire and the interaction of these configurations with UHF built fundamental blocks for the guidewire steering techniques in the MRI scanner. Therefore, we investigated the cardinal configurations where the permanent magnet was placed parallel, perpendicular, and antiparallel to the guidewire tip. Since the strength of the MRI scanner’s main field cannot be changed during the steering, to demonstrate different actuation strategies, we built a robotic MRI platform controlled in the *x* direction and yaw angle (fig. S7). The first steering mode that we investigated is the guidewire steering by rotating the platform of the guidewire relative to the MRI field, similar to (*27*), as shown in Fig. 3A. While this steering mode is relatively impractical since it requires rotating the patient in the MRI scanner, it is essential for a comprehensive understanding of the UHF steering modes. Using a robotic MRI motion stage capable of controlling *x*-*y* and yaw motion, we measured the tip angle for different base angles at 0.5 and 7 T magnetic fields for the parallel configuration (Fig. 3B). We observed that the guidewire could be aligned with the 7 T field with less than 11° at any base angle, while tip angle could reach beyond 45° at 0.5 T for large base angles. These results aligned with our design optimization simulations, where we expect a maximum 80° rotation at 7 T and around 30° rotation range during overall motion. Our results suggested that with the large workspace up to 161° in the guidewire coordinate frame at 7 T (Fig. 3C), we could steer the guidewire at the imaging center of the MRI scanner rather than being limited to the fringe fields (*27*). Moreover, this steering mode could be extended to other magnet configurations to steer the guidewire at different angles (fig. S8).

The second steering mode was moving the platform of the guidewire relative to the MRI’s main field. We investigated three magnetic configurations, and the range of motion during the motorized stage was inserted in the MRI scanner (Fig. 3, D to F). We repeated the experiments with different base angles and recorded the guidewire tip angle (Fig. 3G). As anticipated, the parallel and perpendicular magnets aligned the guidewire tip close to 0° and 90° at 7 T, respectively, and the overall range of rotation was around 30°. Meanwhile, the antiparallel magnet rotated up to 164° and covered a range of motion as large as 64° depending on the initial platform angle. Such a large rotation was possible since the magnet could rotate the guidewire tip up to 100° angles at 0.5 T due to the large initial magnetic torque. Moreover, as expected, the insertion data of the 90° platform angle for parallel and antiparallel showed symmetric character due to the symmetric magnetic torques. Whereas we observed more complex tip angle trajectories for antiparallel configuration due to the different internal moments on the guidewire and the effect of the reversed magnetic gradient force acting on the magnet.

While the first two steering modes were based on magnetic torque, magnetic gradient–based pulling could also be used for steering the guidewire. The concept of assistive magnetic pulling for parallel magnet configuration has been demonstrated in (*27*). We demonstrated the concept in Fig. 3H, where the magnet was oriented in an antiparallel configuration to the field but was parallel to the magnetic gradient. Moreover, we showed that the magnetic gradient of the MRI could also be used to steer the perpendicular magnetic tip. The effect can be seen from the data of perpendicular magnet in Fig. 3G at low fields close to 750 mm distance and maximum gradient region around the 500 mm distance from the center of the MRI scanner. At a low field of 0.5 to 3 T, the magnetic gradient pulling, 2 to 20 T/m, created enough force to counter the magnetic torque; hence, the guidewire with a perpendicular magnet stayed at a 15° angle at low fields. As the guidewire moved in, the increasing magnetic force dominated the magnetic torque, and the guidewire was bent toward MRI at 500 mm; later, the magnetic torque dominated the motion as the guidewire was moved further in the MRI. Last, the competing effect of magnetic torque and force can be used for steering the perpendicular magnet by moving the base of the guidewire around the center of the MRI, as shown in Fig. 3I.

Last, as a standard steering mode for interventionists, the base twist could be used for steering the guidewire in the MRI scanner. Because of the axial symmetry, the base twist did not affect the guidewire with the parallel magnet. However, it was useful for steering in perpendicular and antiparallel configurations in 3D space where gravitational forces caused a negligible effect on the tip position (see note S2) (Fig. 3J**)**. Combining all these steering modes with manual guidewire insertion, we could perform different navigation tasks in the MRI scanner.

### In situ magnetization direction switching at UHF

**Fig. 4.**
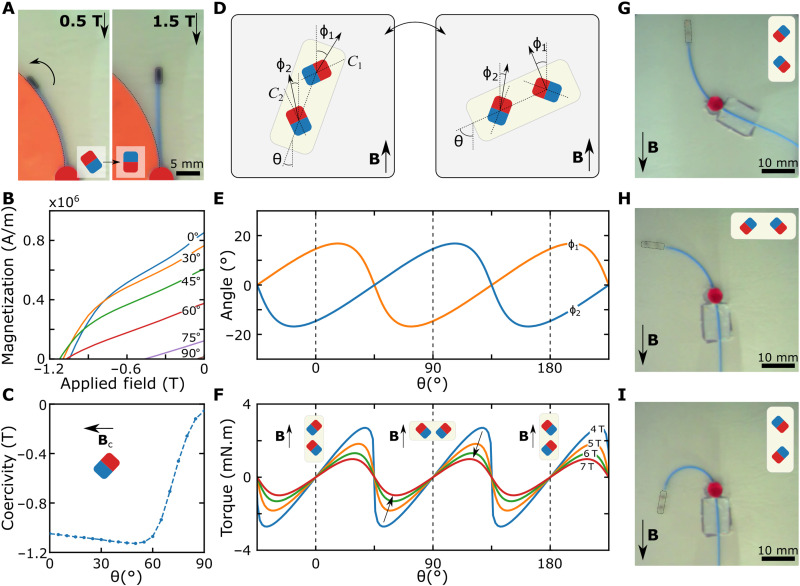
In situ remagnetization method and demonstration at UHF inside constrained spaces. (**A**) Remagnetization demonstration with an antiparallel magnet with the constrained guidewire. An arrow represents the direction of the magnetic torque. The switching magnetic easy axis is shown in the corner. (**B**) The experimental demagnetization curve of the neodymium magnet for different magnet orientations. The angles are shown on the curves. The 90° line passes near the origin. (**C**) The experimental coercivity field at different magnet orientations. The field is applied in the reverse direction to the magnet orientation, as shown in the schematic. (**D**) The dual magnetic tip concept. The magnets are placed at a 45° angle to the long axis of the tip. The switched magnetization direction is shown for parallel and perpendicular configurations. (**E**) The calculated magnetization angles of two permanent magnets for different orientations at 7 T. The magnetization angles are calculated by solving the energy optimization for each magnet. The dashed lines represent the equilibrium points. (**F**) The calculated magnetic torques for different magnet orientations at different magnetic field strengths. The magnetic configuration is shown with the schematics. The field strength is shown on the curves. The arrows show the decreasing magnetic torque due to the increased magnetic field strength. (**G** to **I**) The dual magnetic tip in parallel (G), perpendicular (H), and antiparallel (I) configurations at 7 T. The magnetization direction of the magnets is shown in the schematics.

The last important consideration in the magnetic actuation is the potential of the magnetization direction switch during navigation at UHF. Because of the uniaxial nature of the neodymium magnet, the magnetization direction could be switched through an in situ remagnetization process if the guidewire tip with antiparallel magnetization was physically constrained during insertion into the MRI scanner (Fig. 4A). However, if the guidewire tip was able to rotate over 90° without demagnetization, the remagnetization did not occur. To understand at which magnetic fields in situ remagnetization occurred, we investigated the coercivity field for different magnet angles by observing the second quadrant of the magnetization curve (Fig. 4B). We plotted the coercivity field as a function of the angle in Fig. 4C, which showed a maximum coercivity of 1.1 T at 50° and a minimum coercivity field at 90°. Our result suggests that the magnet would switch its magnetization if constrained during the transition from low field to 1.1 T and above fields. The in situ magnetization switch could be used to switch the magnet configuration during navigation from antiparallel to parallel configurations or to lock the orientation in tubular-constrained structures.

The in situ remagnetization concepts at UHF could lead to an even more interesting magnetic guidewire design with dual stability when two permanent magnets were placed perpendicular to each other at the guidewire tip, as shown in Fig. 4D. Dual stability arose due to the continuous magnetization at UHF and magnetization switching. Therefore, we modeled the magnetization directions of the two magnets perpendicular to each other, considering magnetization switching at 7 T (Fig. 4E). Then, we calculated the total magnetic torque acting on the magnets by the external magnetic field, showing that the superposition of the magnetic torque of two magnets led to stable equilibrium points at 0°, 90°, and 180° (Fig. 4F). However, because of the asymmetry of the superposition of magnetic torques, we observed a decreasing magnetic torque as field strength increased. We demonstrated that the dual stability allowed us to operate the same guidewire in parallel (Fig. 4G), perpendicular (Fig. 4H), and antiparallel (Fig. 4I) configuration shown in Fig. 3 (D to F), which was a unique capability of magnetic actuation at UHF.

### 2D magnetic steering at UHF

**Fig. 5.**
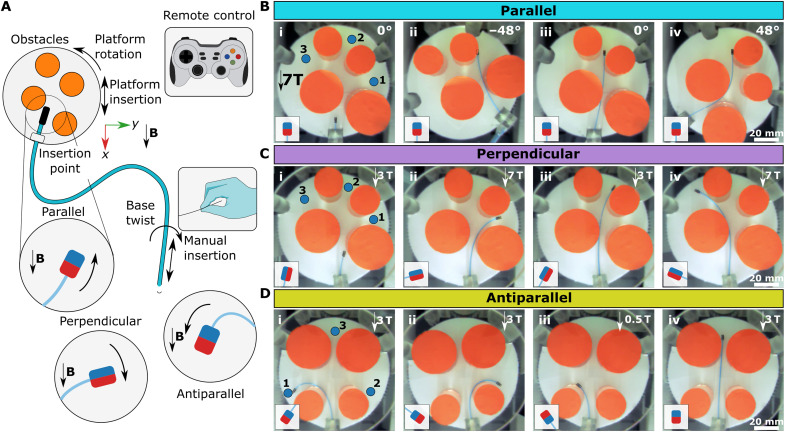
Obstacle avoidance by magnetic steering at UHF. (**A**) Schematic illustration of the obstacle avoidance experimental setup. The obstacles are placed in the robotic platform with remotely controlled motion in the *x* direction and yaw angle. The guidewire is inserted into the platform through the insertion point, and an operator manually controls the insertion and twist of the guidewire by hand at the entrance of the MRI scanner bore. The same platform is used for demonstration with three different magnetic configurations. (**B**) Obstacle avoidance with guidewire with a parallel magnet. The guidewire is steered to three target points shown in (i) using the platform rotation and guidewire insertion at 7 T. The magnetic gradient is used as an assistive force in (iv). (**C**) Obstacle avoidance experiment with a guidewire with a perpendicular magnet. The guidewire is steered to three target points shown in (i) using the platform motion in the *x* direction and the base twist. The operator controlled the bending of the guidewire through the magnetic field strength. The platform angle is kept the same throughout the navigation. (**D**) Obstacle avoidance with guidewire with an antiparallel magnet. The guidewire is steered to three target points shown in (i) using the platform motion in the *x* direction, base twist, and in situ remagnetization. The first two target points are reached using the antiparallel configuration’s larger bending. For the last target point, the guidewire is physically constrained using the obstacles (iii) and remagnetized into the parallel configuration.

We performed steering experiments in the 2D plane with obstacles to demonstrate the capability of UHF magnetic steering for three cardinal magnet configurations. We used the robotic MRI platform to control motion in the *x* direction and yaw angle using a joystick controller, and a human operator manually controlled the insertion and base twist of the guidewire in front of the MRI bore (Fig. 5A). First, we performed obstacle avoidance tasks using parallel magnet configuration at the 7-T isocenter of the MRI scanner to illustrate the difference between FFN and UHF (movie S1). During the task, the experiment setup was robotically rotated through joystick inputs to reach different tip angles with the help of magnetic torques, and the operator did the manual insertion to advance the guidewire to three target regions (Fig. 5B, i). The first target point required a nearly 90° rotation from the starting point, the second target point required simple obstacle avoidance, and the third target point required a large deflection following an obstacle avoidance maneuver. The operator could reach the first target by a −48° platform rotation by leveraging the obstacle as a hinge. The second target was reached by a small obstacle avoidance maneuver within the 10° platform rotation. Last, the last target required a large rotation of 48°, and the platform moved out of the MRI to take advantage of 2.5 T/m assistive magnetic gradient pulling. Next, we repeated the navigation task at the fringe fields where the magnetic field was 0.5 T (movie S2). As we optimized the stiffness parameter of the guidewire for high-field actuation, we needed more platform rotation to reach the first target, and the third target could not be reached at all due to limited magnetic torques. While navigation tasks were successful, we needed to rotate the platform up to 48°, which was impractical in a clinical scenario where the patient has limited space to move in the MRI scanner, except for the cases where local rotation is possible, e.g., head motion. However, the head motion would cause substantial image distortions and a drop in MR image quality that should be addressed in future studies.

Second, we demonstrated the same task with a guidewire with a perpendicular magnet to demonstrate the potential of UHF steering without platform rotation (movie S3). During the navigation, the platform angle was kept fixed, while the platform’s position to the center of the MRI was controlled by joystick input. At the same time, the operator advanced the guidewire by base twist and manual insertion. The operator used the magnetic field strength–depended curvature to steer the guidewire by controlling the platform’s position in the MRI scanner (Fig. 5C). To take advantage of nearly 90° bending, the operator moved the platform to 7 T for the first target. Then, the platform was moved back to 3 T to decrease the bending, and the operator applied base twist and insertion maneuvers to reach the second target. Last, the operator reached the third target by base twist and increasing the bending by moving the platform to 7 T. The magnetic configuration of the guidewire allowed us to operate the guidewire as a curvature-controlled traditional guidewire and reach the targets without requiring platform rotation.

Third, we performed a navigation task using the guidewire with an antiparallel magnet to three target points, as shown in Fig. 5D. The operator should perform over 90° bending to reach two target locations and advance in the forward direction (movie S4). The operator steered the guidewire to the first two targets using the base twist and moving the platform to 7 T for large bending. Later, the guidewire was positioned to be physically constrained. Then, the magnetization was switched to the parallel configuration to reach the last target, demonstrating the potential of magnetization switching in navigation.

### Remagnetization in vascular structures

**Fig. 6.**
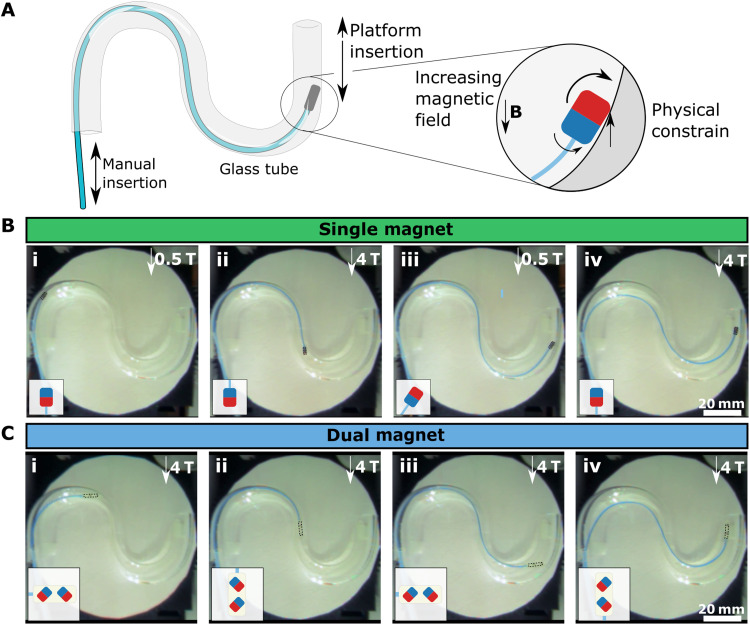
In situ magnetization switch demonstration for orientation locking. (**A**) Schematic illustration of the orientation locking experimental setup. An S-shaped glass tube is used to demonstrate the magnetization switch inside constrained tubular structures. The guidewire is manually inserted by hand, and the platform motion in the *x* direction is controlled remotely. The schematic showed the physical constraining procedure in the tube. (**B**) The magnetization switching with a single parallel magnet. The guidewire is inserted in the tube at a low field of 0.5 T, and the magnetic field is increased by moving the platform in the MRI scanner when the magnet is physically constrained (iii). The state of the magnetization direction is shown in the schematics. (**C**) The magnetization switching with a dual magnet configuration. The snapshots are captured at three different magnetic orientations during the guidewire insertion.

Next, we demonstrated the continuous magnetization switching during navigation in a vascular structure using single parallel magnetic and dual magnet configurations (movies S5 and S6). The guidewires were placed into an S-shaped glass tube and manually advanced in the tube, while the magnetic field was controlled by moving the platform using the joystick input (Fig. 6A). First, the operator advanced the guidewire at a 0.5 T low field at which the magnetic torque was not strong enough to counter the bending of the guidewire. Then, the magnetization was switched by moving the stage in the MRI scanner to apply a 4-T magnetic field on the magnet. The single magnet’s magnetization direction switched two times between the two states (Fig. 6B). In contrast, the dual magnet configuration switched the magnetization state four times between three states during the overall trajectory (Fig. 6C). While the magnetization switching did not contribute to steering in these demonstrations, it locked the orientation of the guidewire tip. As a result, it prevented the guidewire tip from leaning over the tube walls.

### 3D vascular navigation

**Fig. 7.**
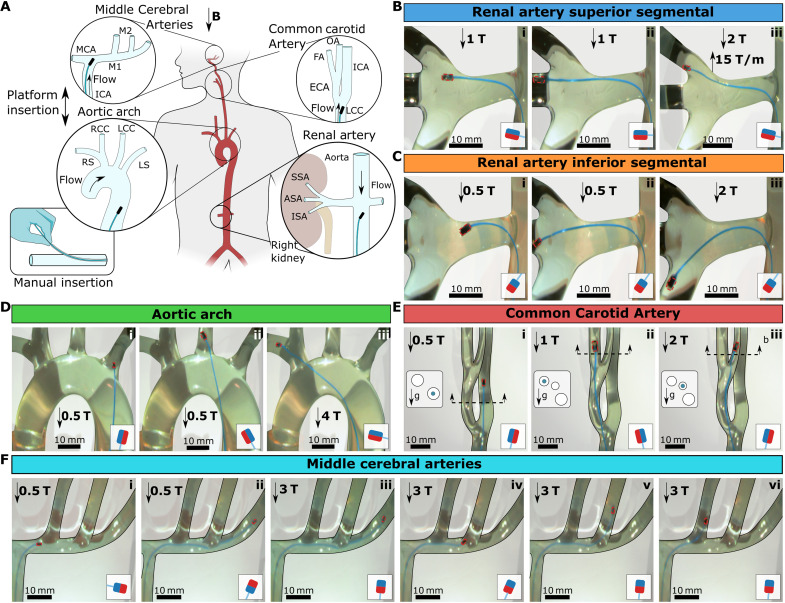
3D vascular navigation under physiological flow. (**A**) Schematic illustration of 3D vasculature phantoms emulating the 3D vasculature. Four different vascular models have been tested: renal artery, aortic arch, CCA, and MCAs. The guidewires in the schematics depicted the insertion directions, and the flow arrows showed the physiological flow’s inlet direction. The guidewire was introduced through a small insertion in the water pipe via a Teflon tube to simulate the groin access, as shown in the bottom left corner. (**B**) Guidewire steering to the superior segmental artery (SSA). (**C**) Guidewire steering to the inferior segmental artery (ISA). (**D**) Guidewire steering to the aortic arch. (**E**) Guidewire steering to the left CCA. The cross-sections of the vascular structure were given on the left of each frame to show the 3D alignment of the tubes and the gravity direction. The blue circles in the cross section show the branch guidewire is located. (**F**) Guidewire steering in MCAs. The magnetic configurations are shown in the small schematic in each frame. Scale bars, 10 mm.

We performed 3D vascular navigation experiments to demonstrate the capabilities of UHF guidewire steering using perpendicular and antiparallel magnet configurations without any platform rotation. To mimic realistic vascular structures commonly targeted in clinical applications (Fig. 7A), we built 3D vascular glass models of renal arteries, aortic arch, common carotid artery (CCA), and middle cerebral arteries (MCAs) (fig. S10). To observe the effect of arterial flow on navigation, we mimicked the blood flow conditions with water using a physiological cardiac flow simulation pump (SuperPump, VivitroLabs, Canada). The guidewire was controlled using manual guidewire insertion and base twist. The robotic MRI stage was controlled via a remote controller in the *x* direction, and platform rotation was not used during the navigation. The guidewire was introduced to the vascular model through a Teflon access port placed on the tubing with a 40 cm distance to the steering target to simulate groin access conditions.

In the first experiment, we targeted the renal arteries and steered the guidewire to the right kidney’s anterior, superior, and inferior segmental arteries (ASA/SSA/ISA). We set the stroke volume to 100 ml per beat during the experiment to simulate flow in the descending aorta. First, we used the guidewire with the perpendicular magnet (Fig. 7B and movie S7). Next, the guidewire was inserted toward the position of renal arteries, and the platform was moved to 1 T to bend the guidewire. Then, the guidewire was steered into the renal artery at a 100° branching angle and 15° elevation angle using a base twist (Fig. 7B, i). It was easier to rotate the guidewire’s left kidney during the maneuver than the targeted right kidney because of the lateral magnetic forces acting on the guidewire in the current radial position in the MRI scanner (see note S3) and the reversed aortic flow created mild instability. The guidewire could be inserted into ASA at its resting orientation (Fig. 7B, ii). Then, we steered the guidewire into SSA by moving the robotic platform in the MRI scanner to 2 T and 15 T/m to take advantage of the magnetic gradient for steering (Fig. 7B, iii). To target the ISA, we changed the guidewire to a guidewire with an antiparallel magnet (Fig. 7C and movie S8). Because of the larger deflection of the antiparallel configuration, we steered into the renal artery at 0.5 T (Fig. 7C, i), and the guidewire could be inserted into ASA at its resting orientation (Fig. 7C, ii). Then, we moved the platform to 2 T to increase bending and inserted the guidewire into ISA with a 45° elevation angle (Fig. 7C, iii).

Second, we performed steering experiments in a type 1 (normal) aortic arch model (*40*) using a guidewire with a perpendicular magnet under 100 ml per beat flow (Fig. 7D and movie S9). We tested navigation into the right/left subclavian (RS/LS) and common carotid (RCC/LCC) arteries. First, the guidewire was steered into the LS at 0.5 T using a base twist, advantaging the flow and artery geometry (Fig. 7D, i). Then, the guidewire was realigned to LCC using base twist (Fig. 7D, ii). Next, we pushed the guidewire forward to maneuver into LCC with reversed curvature while physically performing a base twist to move into LCC. Later, we moved the platform to 4 T to bend the guidewire further for steering into RS (Fig. 7D, iii). We observed that it was not possible to maneuver the guidewire into RCC due to the final resting orientation of the guidewire in RS in the type 1 aortic arch model.

Third, we performed steering experiments in CCA and demonstrated 3D steering capabilities in narrow vessels in internal and external carotid arteries (ECA and ICA) and later into facial and occipital arteries (FA/OA) with a 20 ml per beat stroke volume through the CCA (Fig. 7E and movie S10). With the natural insertion angle to CCA, the guidewire directly moved into the ICA at 0.5 T when inserted (Fig. 7E, i). Next, we moved the guidewire to the ECA/ICA junction and the platform to 1 T to steer into ECA. Then, the guidewire steered into the ECA by the base twist. Again, at the natural insertion angle in ECA, the guidewire moved into FA (Fig. 7E, ii). Because of the narrow junction, we needed a higher field to move into the occipital arteries. Therefore, we moved the platform to 2 T and inserted the guidewire into OA with a base twist. Then, we gradually moved out to the 0.5 T as we inserted the guidewire to the occipital artery to move in the 2-mm-diameter vessel more easily (Fig. 7E, iii).

Last, we performed experiments in an MCAs model mimicking M1 to M2 junction with 3D branching with 20 ml per beat stroke volume through ICA (Fig. 7F). We first tested the guidewire with the perpendicular magnet (movie S11). The guidewire was easily steered into the M1 from ICA at 1 T and entered directly into the proximal M2 branch. However, because of the limited maximum bending of the perpendicular magnet configuration, we could not steer the guidewire into the distal M2 branches. Therefore, to target the distal M2 branches, we changed the guidewire to an antiparallel magnet configuration (movie S12). Compared to the perpendicular magnet, it was much harder to steer the antiparallel magnet into the M1; therefore, we reinforced the guidewire with a 0.9-mm-diameter Teflon tube acting as a catheter at the ICA-M1 junction (Fig. 7F, i). Then, the guidewire was inserted into M1 using a base twist. The guidewire could be inserted until the last M2 vessel due to the tip bending over 90°. To steer into other M2 vessels, we used magnetization switching. The guidewire was first inserted in distal M2 at 0.5 T, and the catheter was advanced up to the guidewire tip to provide additional physical constraint for remagnetization (Fig. 7F, ii). Then, the platform was moved to 3 T, and the tip magnet was switched to the parallel configuration (Fig. 7F, iii). Last, the guidewire was pulled back to the position of the other M2 junctions (Fig. 7F, iv) and inserted into proximal M2 branches with 30° and 0° elevation angles (Fig. 7F, v and vi). It is important to note that if the remagnetized guidewire with parallel configuration was pulled accidentally too much and returned to the ICA, then it is impossible to steer back into the M1. The operator should completely pull out the guidewire from the patient, remagnetize, and reinsert for another trial.

Our results demonstrated the feasibility of 3D vascular navigation in various vascular structures toward and against the flow direction. However, we observed that the lateral magnetic forces at the entrance of the MRI bore, and hydrodynamic forces make it considerably harder to control tip orientation using a base twist. It would be impossible to perform some of the described maneuvers without direct image feedback from the camera. Furthermore, since we cannot use MRI during most of these maneuvers outside of MRI’s field of view, we would need alternative shape-tracking methods in clinical applications.

### Guidewire steering during MRI

**Fig. 8.**
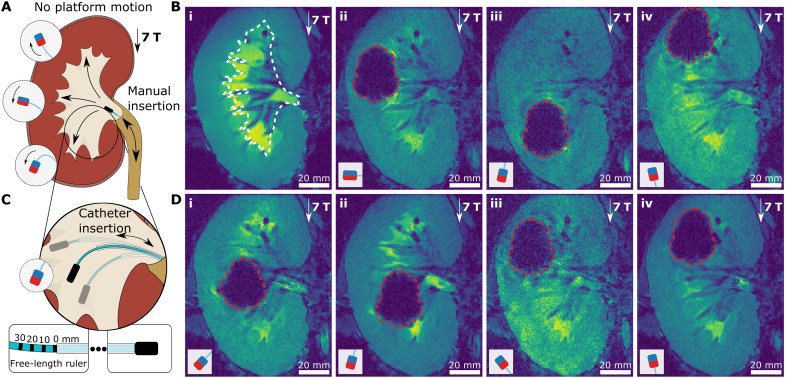
UHF guidewire steering during MRI. (**A**) Schematic illustration of a navigation experiment in a renal cavity of a porcine kidney ex vivo. The kidney was placed at the MRI imaging center, i.e., uniform 7-T magnetic field, and not moved during guidewire steering in the renal cavity. The guidewire was inserted through the urethra. The three target directions and corresponding magnetic tip configurations are shown. (**B**) MR images of guidewire insertion. The approximate boundaries of the renal cavity were shown with white dashed lines on the preoperational MRI image of the kidney (i). Then, the real-time MR image snapshots of the guidewires with perpendicular (ii), antiparallel (iii), and parallel (iv) magnets were shown in the renal cavity. (**C**) Supporting catheter-based steering of the guidewire. The three cases of different guidewire free lengths are depicted for antiparallel magnet configuration. The free-length ruler with the supporting catheter is shown at the bottom. (**D**) MR images of guidewire steering. The guidewire with the antiparallel magnet, as shown in (i) and (ii), with guidewire free lengths of 0 and 20 mm, respectively. The guidewire with the parallel magnet, as shown in (iii) and (iv), with guidewire free lengths of 0 and 20 mm, respectively. The boundaries of the tip magnet MR image artifacts are shown with red dashed lines. The magnetic configurations are shown in the small schematic in each frame. Scale bars, 20 mm.

To demonstrate the potential of UHF magnetic actuation during MRI, we performed guidewire steering experiments in the renal cavity of a porcine kidney ex vivo using guidewires with different magnetic configurations to target different kidney regions (Fig. 8A). We filled the renal cavity of the porcine kidney with the Ringer solution and placed in the MRI scanner. First, we performed a preoperational MRI to visualize the boundaries of the renal cavity (Fig. 8B, i). Then, we inserted guidewires with perpendicular (movie S13), antiparallel, and parallel (movie S14) magnets through the urethra during real-time MRI imaging with 1-Hz fast imaging with steady-state precession (FISP) sequence in the coronal plane. During navigation, the guidewire tip position was monitored using the magnetic distortion caused by the magnet, which was 37 mm in height and 29 mm in width. While we only used MR images for visual monitoring during navigation, the magnetic signature of the magnets at the tip of the guidewire could be used for tracking the exact guidewire tip position in 3D space in the future (*25*, *41*, *42*). First, the guidewire with the perpendicular magnet was inserted, and it could only be steered to the calyx directly in front of the urethra due to the entrance orientation to the renal cavity and magnetic torque (Fig. 8B, ii). Then, the guidewire with the antiparallel magnet is inserted (Fig. 8B, iii). To avoid remagnetization of the tip magnet, we moved the kidney out of MRI, i.e., 0.5 T, and moved it back to 7 T after inserting the guidewire into the renal cavity. Because of the uniform 7-T field at the center of the MRI scanner, the guidewire reached the lower calyx with the maximum bending for which we designed the guidewire. Last, we repeated with parallel magnet configuration to reach the upper calyx (Fig. 8B, iv). While this maximum bending enabled us to reach the upper and lower calyxes using parallel and antiparallel magnet configurations, we had limited steering capacity.

To further demonstrate the UHF magnetic steering capabilities at a constant 7-T field, we introduced a supporting Teflon catheter with 900-μm diameter over the guidewire to control the tip angle by changing the free length of the guidewire tip, as shown in Fig. 8C (see note S4 for the steering model at constant field UHF actuation). By controlling the guidewire free length during insertion, we steered the guidewire with an antiparallel magnet to target positions in the lower part renal cavity (Fig. 8D, i and ii) and the guidewire with a parallel magnet to target positions in the upper part cavity (Fig. 8D, iii and iv). To control the free length of the guidewire, we painted a free-length ruler on the guidewire base and aligned the catheter desired position, as shown in Fig. 8C. We set the guidewire free length to 0 mm in Fig. 8D (i and iii) for minimum bending and 20 mm in Fig. 8D (ii and iv). Moreover, we demonstrated the remagnetization from antiparallel configuration to parallel configuration by constraining the guidewire tip in the urethra with the help of a supporting catheter without moving the platform in movie S1, which enabled us to steer to lower and upper calyces sequentially in a single insertion.

## DISCUSSION

In this study, we introduced fundamental magnetic steering concepts for magnetic guidewires at UHF. Combining the theory of magnetism with continuum mechanics, we established magnetic guidewire design principles maximizing steering capabilities in the MRI scanner. We have illustrated that magnetic guidewires, as simple as precurved guidewires, could be used as an effective intervention tool in the MRI scanner. We demonstrated magnetic steering not only in the fringe fields close to the MRI bore entrance/end but also at the center of the MRI scanner allowing the integration of MRI imaging in the future. Moreover, we expanded the literature on magnetic actuation methods in robotics by elucidating the continuous magnetization processes of uniaxial permanent magnets above magnetic saturation and exploring unique features of UHF, such as magnetization switching and dual stability using uniaxial permanent magnets. Demonstrating these magnetic actuation concepts in the UHF, we provided building blocks for future design opportunities for magnetic medical robotics.

Although we have focused on fundamental actuation modes and manual actuation of the guidewires, the demonstrated actuation techniques could be used for more complex guidewire designs and robotic actuation mechanisms. For instance, we demonstrated preliminary results of a supporting catheter extending steering capabilities under uniform UHF. Such combined actuation mechanisms could be used in the future to create robot designs merging concentric tube actuation (*13*) and the UHF actuation. Similarly, the guidewires with multiple magnets in different configurations throughout the length could mimic the shape of precurved guidewires (*34*, *38*), or multiple guidewires with different magnetic configurations could be used in a catheter shaft to create local curvature without sacrificing the operational comfort of the interventionist. Moreover, UHF magnetic steering could be coupled with the other robotic guidewire actuation mechanism available in MRI scanners, such as Lorentz force–based actuation (*17*, *18*), to benefit their advantages while eliminating the drawbacks. For example, a dual stable magnetic tip could provide intermediate stable states for Lorentz force–based actuation to prevent heating problems during long actuation durations.

Besides the potential design benefits of UHF actuation in robotics, there are still issues to tackle regarding the medical usage of the proposed guidewires. First, the miniaturization of the magnetic tip is crucial and necessary to operate in the human vasculature system. While the guidewire with a 500-μm diameter is comparable to the commercial guidewires, the magnetic tip is relatively large, especially for perpendicular and dual magnet configurations, due to the unavailability of non-axially magnetized commercial cylindrical magnets. This issue could be overcome by manufacturing custom permanent magnets; however, it is an admissible manufacturing challenge. A smaller magnet would also decrease the size of the MR image artifact and ease monitoring the surrounding tissue. Another issue is modifying the guidewire design according to the field strength of the clinical MRI scanners. Because of availability, we designed and tested our guidewires in a preclinical small-animal 7-T MRI scanner. However, the 7-T human MRI scanners are also becoming available in clinics (*43*), and the proposed method can be directly used in these MRI scanners. Moreover, the proposed guidewire design principle could be extended to typical lower-field clinical human MRI scanners, such as 1.5 and 3 T. The guidewire would still operate in saturation regime at these field strengths for angles smaller than 30°, but we must compensate for the magnetic torque decrease by either increasing the tip magnet’s size or decreasing the guidewire’s overall stiffness. Moreover, the magnetic torque–based steering could also be adapted to vertical open-bore MRI scanners by reconsidering the magnetic field direction of the MRI scanner and the magnetic configuration of the guidewire.

Furthermore, there are certain clinical challenges and limitations for UHF actuation. For example, one major limitation is that the UHF magnetic actuation (and any other existing MR-guided actuation) could not be used on patients with ferromagnetic implants since it would apply enormous magnetic torque and force on the implants, most likely leading to implant dislocation and severe injury. Similarly, physicians should also pay extra attention while operating on patients with larger conductive nonmagnetic implants like leg implants. Besides eddy current heating problems associated with the conductive implant, moving in/out of the high nonuniform magnetic field would create a substantial eddy current drag force that could dislocate the implant. However, operating at a low platform speed could avoid such complications. Moreover, the platform’s speed could also cause dizziness to the patient. Versluis *et al.* (*44*) reported that 34% of patients experienced dizziness when moved into a 7-T MRI scanner at 7 cm/s speed, which is close to the 5 cm/s platform speed we used in this study. While UHF magnetic actuation is not limited to 7-T MRI scanners and could be performed at 1.5 and 3 T as well, it is important to study the effect of the motion in/out of the high magnetic fields on patients for future clinical applications.

Last, the most important milestone in the future medical application of the UHF magnetic guidewire steering strategies is the localization of the guidewire. In 3D vascular navigation, we observed the importance of visual feedback in complex maneuvers. Using a supporting catheter, we illustrated the feasibility of UHF magnetic guidewire steering during MRI imaging. However, the steering capacity at the uniform field of the MRI scanner was limited, and the true strength of UHF field actuation strategies comes from controlling the field strength acting on the guidewire tip. Therefore, it is important to have auxiliary methods to track the guidewire out the size of the field of view of the MRI scanners. One potential solution would be integrating different imaging modalities that could operate in the MRI scanner, such as x-ray and ultrasound (*27*, *41*). However, such integration would cause substantial engineering challenges and setup costs. Another solution would be using fiber optic shape sensing methods. For example, fiber Bragg gratings could be integrated into the guidewire design without compromising the MRI compatibility of the guidewire to track the guidewire shape during motion out of the MRI’s imaging center (*45*). Once combined with medical imaging and shape-tracking methods, our UHF actuation methods would greatly affect clinical scenarios as an effective MRI intervention method and pave the way for new magnetic robotics concepts for medical operations.

## MATERIALS AND METHODS

### Cosserat rod model for magnetic actuation at UHF

A discretized Cosserat rod model with magnetic actuation was implemented to simulate the magnetic guidewire in free space, adapting the partial differential equation formulation described in (*46*). In the Cosserat rod model, the state of guidewire was described as **Y** = [**y**_0_^T^, **y**_1_^T^, …, **y**_N_^T^]^T^ and **y**_i_^T^ = [**p**_i_, **R**_i_, **n**_i_, **m**_i_], where **p**_i_ and **R**_i_ were the position and rotation matrix of each segment and **n**_i_ and **m**_i_ were internal force and moment in guidewire’s local frame and *N* was chosen as 20. To apply the magnetic force and torque to the tip of the guidewire, we introduced the platform position and orientation as input to the simulation. For each time step, the simulation calculated the magnetic field and the gradient experienced by the magnet for the corresponding position and orientation of the platform in the MRI scanner. Then, the magnetization direction of the magnet was found by optimizing the energy term in Eq. 2, magnetic torque was calculated by Eq. 5, and magnetic force in the axial direction of the MRI scanner was calculated using Eq. 6. The magnetic force and torque were transferred to the guidewire’s local coordinate frame. They were added to the Cosserat rod model as boundary conditions by defining quadratic cost function (***n***_N_ − ***F***_m_)^2^ + (***m***_N_ − **τ**_m_)^2^, forcing the simulation to equate the tip force and torque to calculated magnetic force and torque, similar to (*18*).

### Magnetization characterization

The magnetization curves of the neodymium permanent magnets were measured using a vibration sample magnetometer (VSM) (MicroSense, Lowell, MA) with maximum 1.8-T magnetic field strength. We printed a special holder aligning the easy axis of the magnet with the measurement axis of the VSM using a stereolithography 3D printer (Form3, Formlabs). The magnet samples were placed in the sample holder and fixed with ultraviolet (UV)–cured 3D printing resin to prevent rotation under high magnetic torque during VSM measurements. Later, the holder was fixed to a glass connector and placed inside the VSM. The sample’s easy axis was first aligned to the VSM, and the sample’s magnetization curve was measured with 0.05-T increments from −1.8 to 1.8 T for different angles with 15° increments. The measurements started from 0 T to capture the hysteresis loop during the ramp-up and down of the magnetic field. Before each magnetization curve measurement, the sample was aligned back to 0° and magnetized in its easy axis to eliminate the history of consecutive measurements affecting each other. We repeated magnetization curve measurements for cylindrical and spherical magnets for saturation magnetization and anisotropy constant measurements. Last, we measured the magnetization curve only in the first quadrant between different starting and ending magnetic fields to observe the effect of the hysteresis.

### Magnetic actuation setup in the MRI scanner

A robotics actuation system was built to demonstrate UHF magnetic actuation in 7-T preclinical small animal MRI (Biospec30/70, Bruker, Germany). The actuation system comprises three control DoF (fig. S7B): a motorized stage (Autopac, Bruker, Germany) controlling insertion of experiment platform to MRI scanner and two piezo actuators (LR23-80, Piezomotor, Sweden) with custom-made nonmagnetic encoders controlling yaw and lateral motion. In addition, an MRI-conditioned eye-tracking camera was placed on top for visual feedback in the MRI scanner. The overall system was MRI-conditioned; however, it was not MRI compatible, i.e., it cannot be used during MRI. The actuation system was integrated into the robotics operating system for remote control. Later, the magnetic actuation system is used for mapping the magnetic field and the gradient in the MRI scanner. A gaussmeter with a 30-T probe (LakeShore Cryotronics, Ohio, USA) was placed on the stage. The magnetic field was sampled with 2.5-mm spacing over the axis of the MRI, and the gradient was calculated by numerical differentiation. For the 2D magnetic field measurements, we measured the magnetic in the axial direction of the MRI scanner in the *x-y* plane. Then, we calculated the gradient of *B_x_* in the *x* and *y* directions with numeric differentiation and the gradient of *B_y_* using the Maxwell equation with no current assumption. Last, we calculated *B_y_* by numerical integration using symmetry and by assuming zero radial magnetic field at the MRI’s axis.

### Guidewire manufacturing

The guidewire prototypes were manufactured using a fiber optic glass rod with a 200-μm-diameter core and 500-μm coating diameter (FP200URT, Thorlabs) and 1-mm-diameter and height neodymium permanent magnets (Magnethandel, Germany). The fiber optic rod was chosen because of its MRI-compatible nature and elastic properties close to commercial guidewires. We measured the effective Young modulus of the system using the small deflection theory (fig. S13A). Five fiber optic rod samples with 3-cm length and 500-μm diameter were deformed for 3 mm. The forces were measured using a force sensor (LSB200, Sensocon, Germany), and the mean effective Young modulus was calculated using *E* = *FL*^3^/3 δ*I*, where *I* = π*r*^4^/4. Later, the magnets were fixed to the fiber optic guidewires using guidewire tips 3D printed using a stereolithography 3D printer (Form3, Formlabs). The tips were designed to have cavities aligned in different directions to allow guidewires with different magnetic configurations. The magnets are tight-fitted in the cavities and sealed with UV-cured 3D printing resin (fig. S13B). Last, the guidewire tips were assembled to guidewires using UV-cured resin. As an extra step in the dual magnet tip, we first fixed one of the magnets using UV-cured resin and placed the second magnet later since the magnets interacted with each other (fig. S13C).

### Magnetic resonance imaging

We used fast FISP sequence navigation experiments in the porcine kidney. We used a 120 mm by 120 mm field of view with 0.93-mm pixel size, 1-mm slice thickness, TE/TR = 2.5/5.0 ms, and 60° flip angle imaging parameters. The total duration of the single imaging was 686 ms, i.e., 1.45-Hz feedback rate. During guidewire steering, the MRI sequence was performed continuously.

### Statistical analysis

All quantitative values were presented as means **±** SD. Student’s *t* test was used for the statistical analysis, and statistical significance was set at a 95% confidence level for all tests (*P* < 0.05).

## Supplementary Materials

### This PDF file includes:

Note S1 to S4 Figs. S1 to S13 Legends for movies S1 to S14 References
