# Supplementary material for: Magnetic guidewire steering at ultrahigh magnetic fields
Source: Sci Adv. 2023 Apr 26;9(17):eadg6438. doi: 10.1126/sciadv.adg6438 (PMC10132757; doi:10.1126/sciadv.adg6438)
Supplement: Supplementary file 1 — Note S1 to S4 Figs. S1 to S13 Legends for movies S1 to S14 References [file sciadv.adg6438_sm.pdf]

Science Advances
AAAS

Supplementary Materials for

Magnetic guidewire steering at ultrahigh magnetic fields

Mehmet Efe Tiryaki *et al.*

Corresponding author: Metin Sitti, sitti@is.mpg.de

Sci. Adv. **9**, eadg6438 (2023)
DOI: 10.1126/sciadv.adg6438

The PDF file includes:

Note S1 to S4
Figs. S1 to S13
Legends for movies S1 to S14
References

Other Supplementary Material for this manuscript includes the following:

Movies S1 to S14

Supplementary Note S1. Magnetic torque comparison for crystal and shape anisotropy

The concept of using a shape anisotropic soft ferromagnetic tip as an alternative to a permanent magnetic tip has been investigated briefly in (27). Azizi *et al.* proposed a stainless steel spring placed into the tip of the guidewire and qualitatively demonstrated potential for utilizing the magnetic gradient forces of MRI's fringe field. We quantitatively compared magnetic torques generated by materials with crystal and shape anisotropy to choose the best magnetic tip material for UHF actuation. To quantify the magnetic torque acting on the soft magnet, we used the continuous magnetization model at the saturation regime described by Abbott *et al.* for ellipsoidal soft magnetic bodies (28). We assumed that the soft body does not have any crystal anisotropy. We rewrote the energy optimization for soft magnets in our convention as follows:

$$E = \min_{\phi} \frac{1}{2}\,(n_r - n_a) M_s^2\, sin^2(\phi - \theta) - M_s H cos(\phi), \tag{S1}$$

where n_r and n_a are the demagnetization factors for the general ellipsoidal shape (*28*). From energy optimization, we could immediately see that the main difference in uniaxial crystal anisotropy in equation 1 and shape anisotropy is the K_1 and (n_r -n_a) $M_s^2/2$ terms, which also reflected the maximum torque calculated reported in (*28*), $\tau_{max} = \mu_0 V (n_r - n_a) M_s^2/2$. In a theoretical maximum, for a soft magnet with an infinite aspect ratio (n_r -n_a) converges to ½, and we can write an absolute maximum magnetic torque for a soft ferromagnetic material as $\tau_{max} = \mu_0 V M_s^2/4$, where saturation magnetization directly define the maximum torque. To use as a reference soft ferromagnet, we measured the saturation magnetization of chromium steel (AISI 52100) which has a high saturation magnetization of 1.49×10^6 Am (fig. S4A). We calculated the absolute maximum torque as $0.56\mu_0 V$, which is the 24.5% of the maximum magnetic torque we observed in neodymium permanent magnet.

Later, we expanded our analysis to more realistic aspect ratios, α. First, we calculated the angle magnetization angle by solving the energy optimization for aspect ratios 2, 5, and 100 as a function of easy axis angle at 1 T, the latest as a limit case (fig. S4B). Even with a relatively low external field of 1 T, the magnetization vector has aligned with the field with less than 30°. Second, we calculated the magnetization angle and magnetic torques for aspect ratio 5 for different field strengths using the same volume of our cylindrical permanent magnet (fig. S4C-D). We observed that even with a relatively large aspect ratio, we could only apply 20% of the magnetic torque generated by the permanent magnet. We could still design a guidewire with parallel configuration using the shape anisotropy-based magnetic torque by using a softer material for the guidewire core; however, the magnetic torque difference between 7 T and 1 T decreased significantly, which would result in a smaller rotation range. Moreover, the magnetic guidewire tip's perpendicular and antiparallel magnetic configuration could not be built with a soft magnetic tip due to geometric limitations and low coercitivity.

Supplementary Note S2. Effect of gravity on 3D actuation

In our simulation environment, we observed a negligible effect of gravity on the final shape of the guidewire since the gravitational forces were relatively small compared to the magnetic force/torque on the tip magnet and the elastic force/torque. We considered two cases to provide a quantitative comparison of gravitational forces and magnetic forces. In the first case, we have a guidewire with a parallel magnet aligned in the field direction, where magnetic torque and force are counteracting the tip magnet's weight and the guidewire's distributed weight, as shown in fig. S9A. Since the guidewire deflection due to gravitational force is very small compared to the magnetic torque, we used a small deflection approximation and neglected the contribution of the magnetic pulling force F_m because of the very small moment arm. Then, we could model the final tip deflection (δ) and tip angle (θ) by superposing the effects of tip force, distributed force, and tip torque shown in fig. S9B (47). We calculated the deflections for the 50 mm free length of the guidewire. The weight of the tip magnet, W_t, was 57.7 μN neglecting the 3D printed resin magnet holder and the distributed force over the guidewire, w_w, was 50 μN/mm. We first calculated total deflection and tip angle due to the contribution of gravitational force, $\delta_g = \delta_w + \delta_t = 2.1$ mm and $\theta_g = \theta_w + \theta_t = 0.058$ rad. Since the magnetic torque is a function of the tip orientation, we looked for a small angle magnetic torque expression by solving equation 2. We took the derivative with respect to ϕ and equated it to zero as follows,

$$K_1 \sin(2(\phi - \theta)) + M_s\, H \sin\phi = 0. \tag{S2}$$

We replaced sin(x)=x using a small angle approximation for ϕ and $2(\phi - \theta)$ and obtained

$$2K_1\phi - 2K_1\theta + M_s\, H\phi = 0. \tag{S3}$$

Then, we wrote ϕ as a linear function of θ,

$$\phi = \frac{2}{2+\frac{M_s H}{K_1}}\theta. \tag{S4}$$

Lastly, we inserted ϕ into equation 5 using small angle approximation and obtained magnetic torque as a linear function of θ,

$$\tau_m(\theta) = \frac{2\mu_0 V M_s H}{2+\frac{M_s H}{K_1}}\theta. \tag{S5}$$

Using the linear torque equation, we can now calculate the final tip orientation by solving the linear relation $\theta = \theta_g - \theta_m$, where θ_m is the rotation contribution of the tip torque $\tau_m(\theta)$. The final tip orientation is then

$$\theta = \frac{\theta_g}{1+\frac{2\mu_0 V M_s H l}{(2+\frac{M_s H}{K_1})EI}}\,. \tag{S6}$$

We calculated the final tip orientation θ at 1.5 T field as 0.006 rad (0.34°) and further decreases at higher magnetic fields. Then we calculated the tip deflection contribution of magnetic torque as 1.3 mm and obtained a final deflection tip, δ, as 0.77 mm for a 50 mm guidewire segment, demonstrating a negligible effect of the gravity.

In the second case, we have a guidewire with a perpendicular magnet configuration where the guidewire is bent in 3D space to align with the magnetic field (fig. S9C). Due to the large nonlinear deflection, we cannot use the cantilever beam model to estimate the beam shape; however, we could provide an approximation shape using the constant curvature model (fig. S9D), assuming the magnetic torque is the order of magnitude larger than other torques acting on the guidewire. Then the bending radius could be written as:

$$\rho = \frac{EI}{\tau_m(\theta)}. \tag{S7}$$

Using the ratio of arch length to radius $\frac{l}{\rho} = \frac{\pi}{2} - \theta$ and linear magnetic torque equation S5, we could calculate the final magnet angle as follows

$$\theta = \frac{\pi}{2\left(1+\frac{l}{EI}\frac{2\mu_0 V M_S H}{\left(2+\frac{M_S H}{K_1}\right)}\right)}, \tag{S8}$$

which gives us θ =10.42° for a 1.5 T magnetic field and 50 mm long guidewire segment, and the corresponding magnetic torque is 9.6×10^{-5} Nm. Then using the constant curvature shape of the guidewire, we could calculate the moment of gravitational forces on the fixed end of the guidewire,

$$\tau_g = \int_0^{\frac{\pi}{2}-\theta} w_w r^2 \sin(\Theta) d\Theta + W_m r sin(\frac{\pi}{2} - \theta) \tag{S9}$$

as 7.3×10^{-6} Nm, which is more than the order of magnitude smaller than the magnetic torque. Lastly, we could approximately calculate the additional tip deflection due to the gravitational force using equation S5 as 0.84°. Therefore, we could see that the shape has minimal effect on the shape of the guidewire. Moreover, this analysis could also be extended to antiparallel magnet configuration without loss of generality. Finally, the gravitational force also creates a twist on the guidewire that is counteracted by torsion in the guidewire and the base twist torque, τ_b, as shown in fig S9E. However, this twisting torque is very small, and the next section explains that radial magnetic forces dominate torsional dynamics.

Supplementary Note S3. Effect of radial magnetic forces on 3D actuation

Since the radial component of the magnetic force is much less compared to the axial component, we have neglected this component in our simulations, where we calculate the shape of the guidewire in 3D free space. However, during vascular navigation experiments, we also observed the radial force's effect during the guidewire's base twist-based steering. Considering the base twist actuation in free space where the fixed end of the guidewire is aligned with the axis of the MRI scanner (fig. S11A), the radial magnetic force will be aligned with the fixed end; hence we will not apply any torsion on the guidewire. However, when the guidewire is off-centered in the MRI bore, the radial magnetic force will have a small moment arm and apply torsion on the guidewire. As a result, a stable axis occurs, as shown in fig. S11B. A similar phenomenon also occurs when the guidewire is off-centered to the MRI scanner in vascular structures, as shown in fig. S11C. Although the operator could observe a significant resistance during base twist action, it is still possible to steer the guidewire. The effect of the radial forces could, in principle, be modeled in future work; however, even with state-of-the-art modeling methods (48), it would be an admissible modeling challenge due to the complex interaction between the guidewire and vessel walls throughout the guidewire body.

Supplementary Note S4. Uniform magnetic field guidewire steering with free-length control

A special case of UHF magnetic actuation is under a uniform ultrahigh magnetic field. At the center of the MRI scanner, we have a uniform magnetic field that would align the guidewire tip to the maximum bending for which we designed and limits our steering capabilities. However, we could provide additional steering capacity to our guidewire by introducing a supporting catheter to control the guidewire's free length, as shown in fig. S12A. Neglecting the effect of gravitational forces, we only have magnetic torque acting on the guidewire tip; therefore, we could use a constant curvature beam model to estimate guidewire shape, where the guidewire and catheter segments would have different radii of curvature (fig. S12B). The radius of the guidewire-only part could be calculated as $\rho_g = \frac{E_g I_g}{\tau_m(\theta)}$, where E_g and I_g are the elastic modulus and moment of inertia of the guidewire, and the radius of the composite part could be calculated as $\rho_c = \frac{E_g I_g + E_c I_c}{\tau_m(\theta)}$, where E_c and I_c are elastic modulus and moment of inertia of the catheter, assuming rigid body interaction between the guidewire and outer Teflon catheter. Then, using linear magnetic torque approximation and the ratio of arch length to the radius of curvature, i.e., $\frac{l - l_g}{\rho_c} = \theta_c$ and $\frac{l_g}{\rho_g} = \frac{\pi}{2} - \theta - \theta_c$, we could calculate the guidewire tip orientation as follows:

$$\theta = \frac{\pi}{2\left(1 + \left(\frac{1 - r_g}{E_g I_g + E_c I_c} + \frac{r_g}{E_g I_g}\right) l \frac{2\mu_0 V M_S H}{\left(2 + \frac{M_S H}{K_1}\right)}\right)}, \quad \text{(S10)}$$

where r_g is the ratio of guidewires free length to total length, l_g/l. Equation S10 converges to equation S8 when the r_g is 1; hence we have the maximum bending (fig. S12C). When the r_g is 0, we have a minimum bending angle (fig. S12D):

$$\theta_{min} = \frac{\pi}{2\left(1+\frac{l}{E_g I_g + E_c I_c}\frac{2\mu_0 V M_S H}{\left(2+\frac{M_S H}{K_1}\right)}\right)}. \quad \text{(S11)}$$

Then we can calculate the guidewire shape by substituting θ back to magnetic force and constant curvature model. While it is possible to estimate the shape in 3D free space, more comprehensive models are needed in the future to predict the shape with contact forces. Moreover, due to the large stiffness of the supporting guidewire, the tip magnet's magnetization direction could exceed small angles, and we might need to solve the nonlinear energy optimization problem to calculate the shape.

Figures

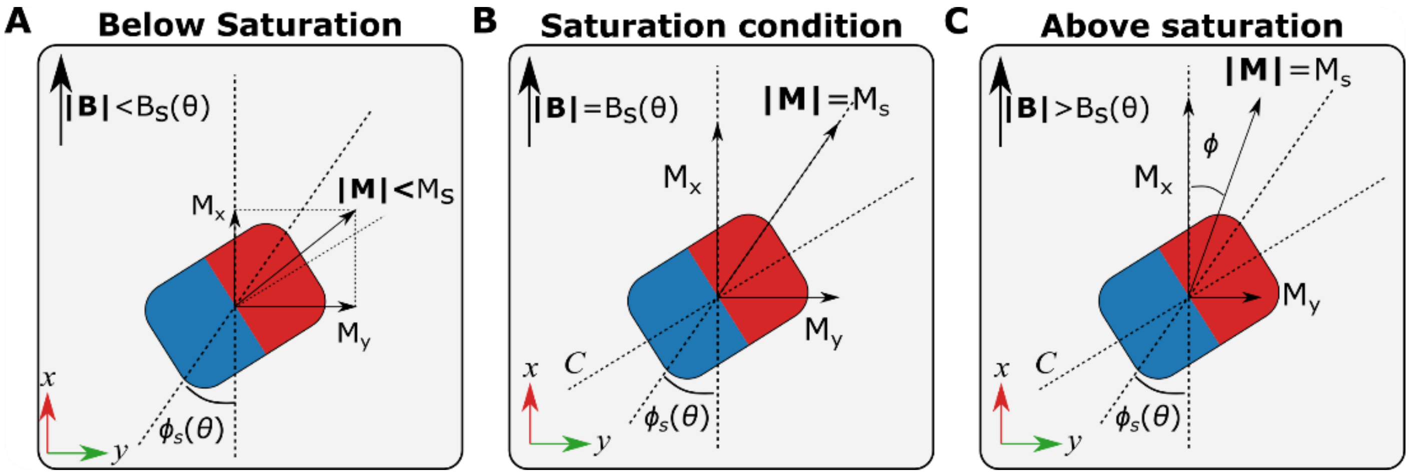

Figure S1. A uniaxial permanent magnet in different magnetic actuation regimes. A) Below saturation. The magnetic field increased the magnetization in the external field direction. The magnetization perpendicular to the external field is assumed to stay constant before saturation. **B)** The saturation condition. The magnetic field reaches a value where the magnitude of the magnetization is equal to the saturation magnetization. **C)** Above saturation. The magnet is totally saturated; hence the magnetization vector starts rotating towards the external magnetic field. The magnetization in the *y* direction decreases while the *x* direction increases and the total magnetization stays the same.

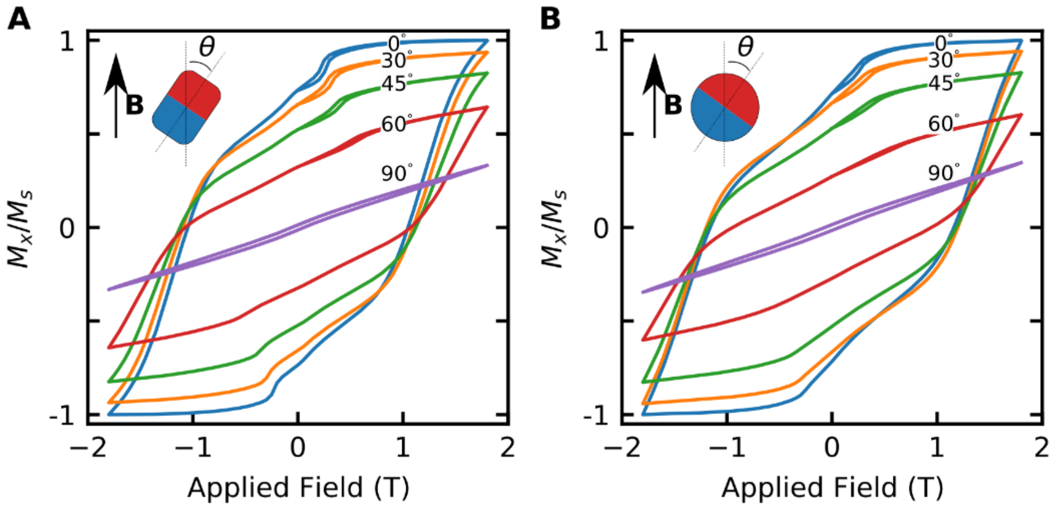

Figure S2. The hysteresis loop comparison for cylindrical magnet and spherical magnet. A) The hysteresis loop for a cylindrical magnet at different magnet orientations. **B)** The hysteresis loop for a spherical magnet at different magnet orientations. The magnet orientations to the applied external field were shown with the schematics.

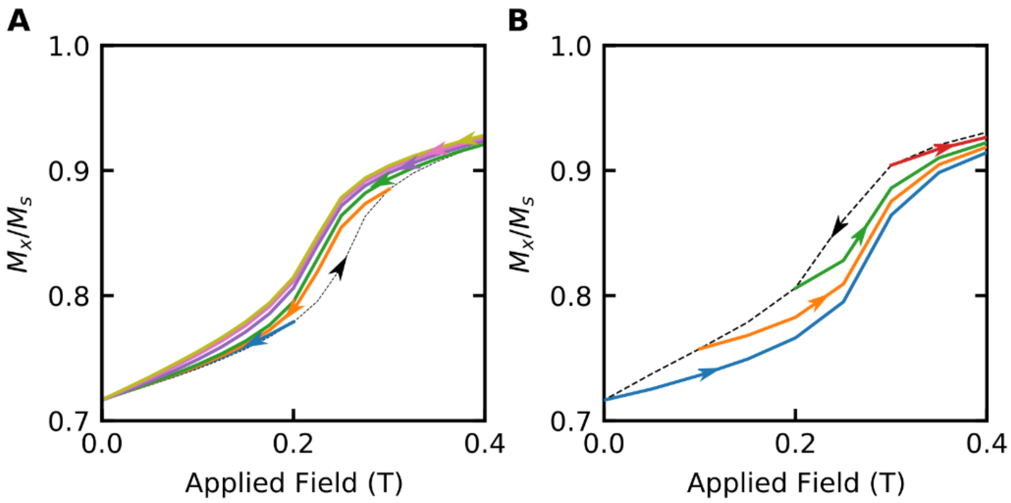

Figure S3. The low field hysteresis behavior of the cylindrical neodymium magnet at *θ=0*. A) Hysteresis during decreasing magnetization. **B)** Hysteresis during increasing magnetization. The magnetization curve was repeatedly upward and downward by changing starting points with 100 mT increments.

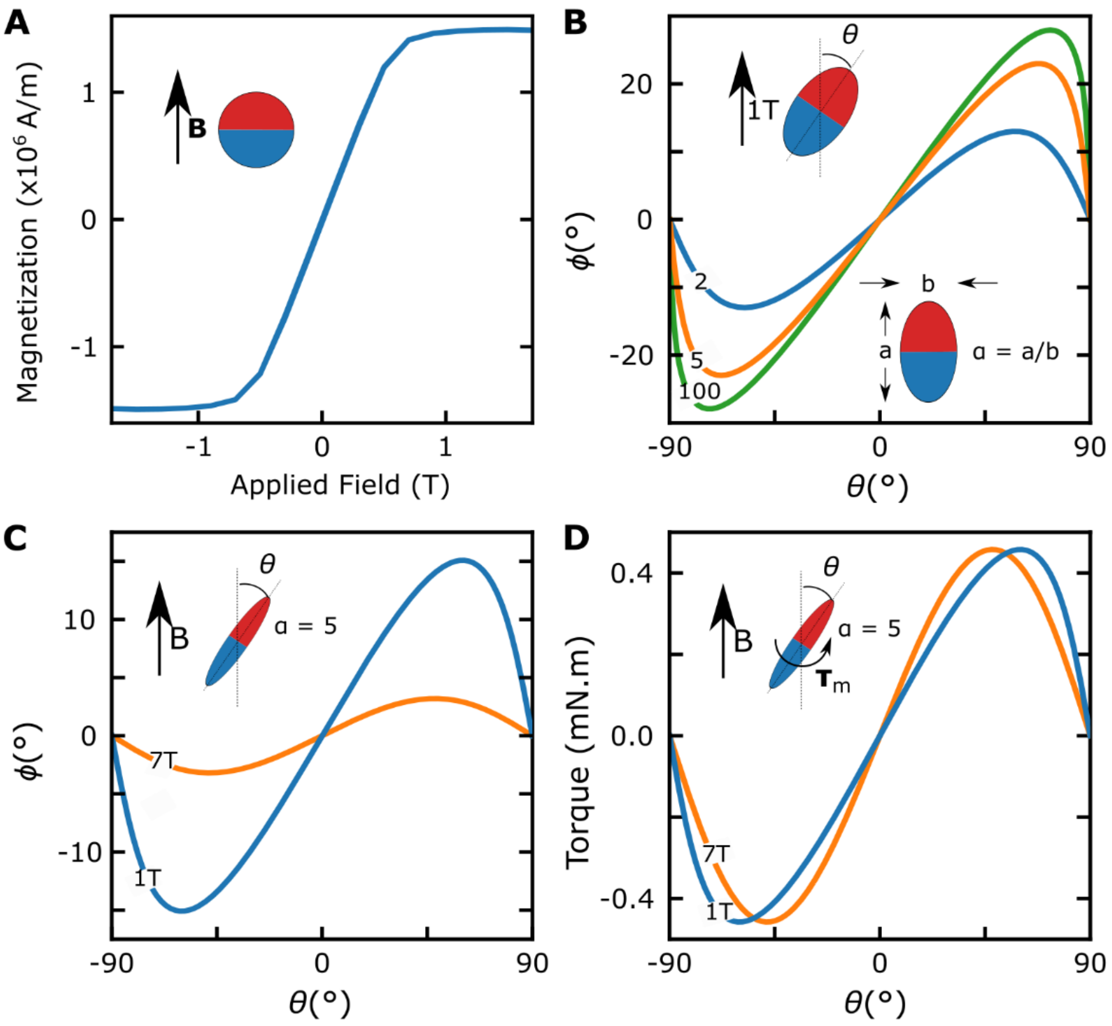

Figure S4. The magnetic torque acting on soft magnets at UHF. A) The magnetization curve of the chromium steel sample. Due to the extremely low coercivity, there is virtually no hysteresis. The saturation magnetization was measured as 1.49×10^{6} Am. **B)** The magnetization angle as a function of soft magnet direction for different aspect ratios at 1 T. The aspect ratio was defined as shown in the schematic. The magnetization angle was measured using energy optimization using shape anisotropy energy for three different anisotropies. The aspect ratio of 100 was used as an upper limit example. **C)** The magnetization angle as a function of magnet direction for different field strengths for aspect ratio 5. **D)** The magnetic torque acting on the soft magnet with an aspect ratio of five at different UHF.

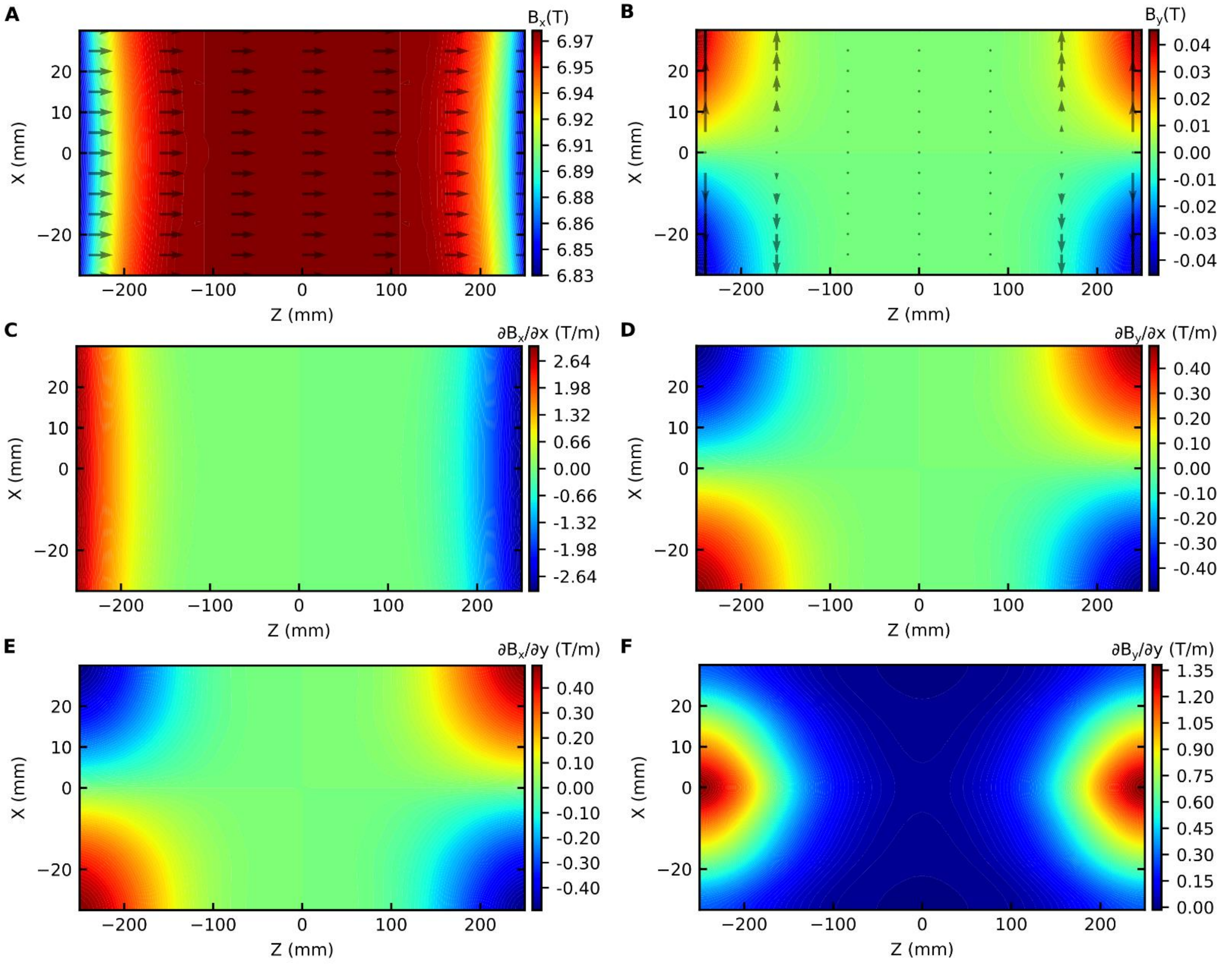

Figure S5. 2D magnetic field measurements in the center of the MRI scanner. A-B) The magnetic field in the *x* and *y* directions. **C-F)** The magnetic field gradients at the *x* and *y* directions.

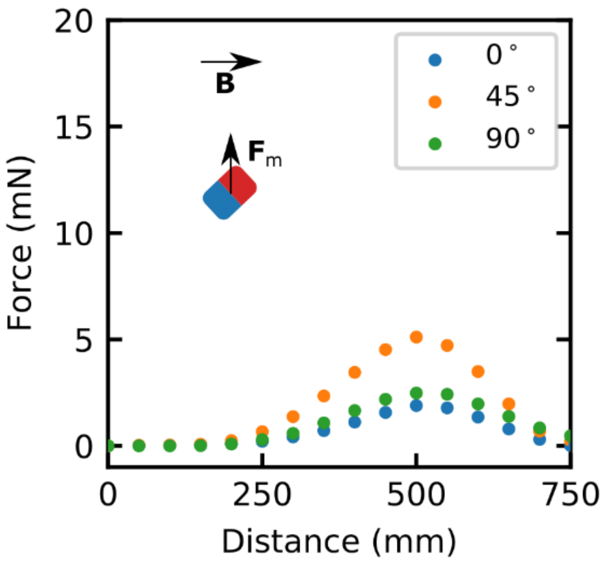

Figure S6. The magnetic gradient in the *y* direction in the MRI scanner for different angles. The dots represented the measurement collected using MRI conditioned force sensor in the y direction. The schematic showed the force direction.

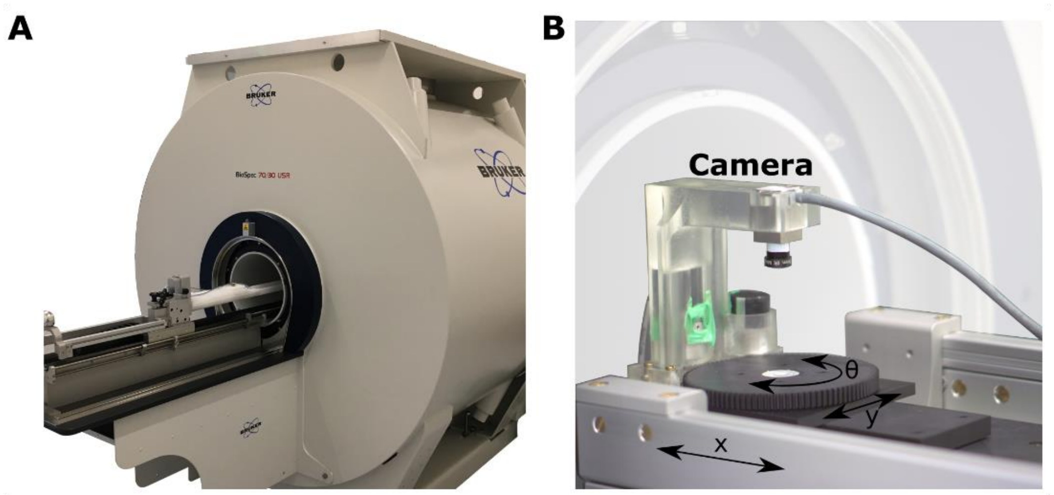

Figure S7. The magnetic actuation system in the MRI system. A) Photo of the 7-Tesla preclinical small-animal MRI scanner. **B)** The robotic motion platform for remote actuation. The platform comprised a linear MRI stage moving in the *x* direction and a custom-made piezo stage controlling motion in the y and yaw direction. In addition, an MRI-conditioned camera was placed to monitor the motion.

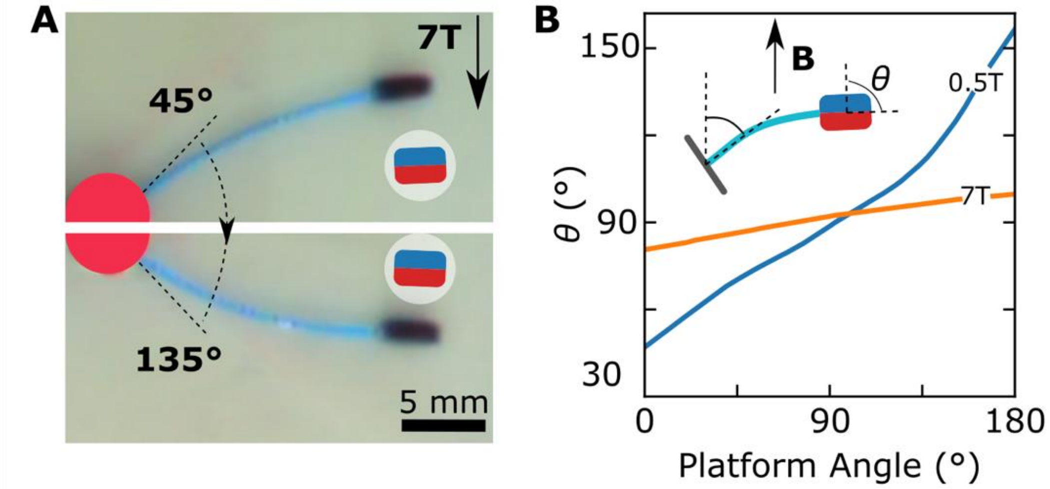

Figure S8. Guidewire with a perpendicular magnet was steered with a motion platform rotation. A) Experiment image of the guidewire with perpendicular magnetic at two different platform angles at 7 T. The magnet schematic shows the magnet direction. **B)** The guidewire tip orientation for different platform angles at 0.5 T and 7 T.

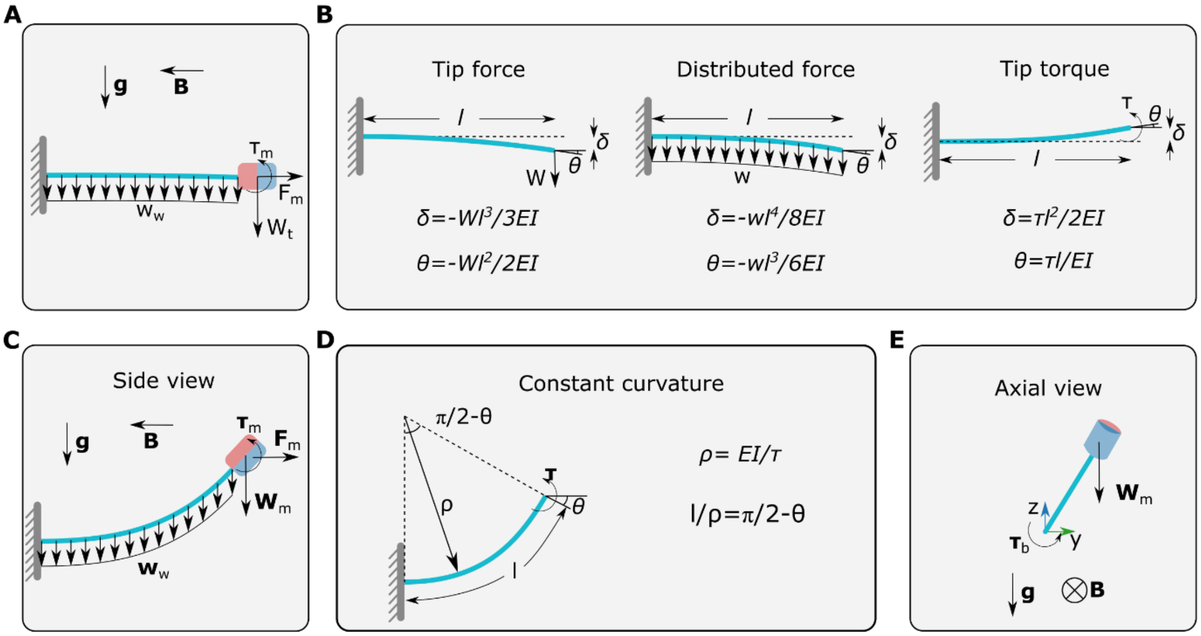

Figure S9. Effect of gravitational forces. A) Forces and torque acting on guidewire with the parallel magnet. The gravitational forces are added as distributed body forces on the guidewire body, w_w, and tip magnet weight, W_t. **B)** The small deflection cantilever beam models for superposition. **C)** Forces and torque acting on guidewire with the perpendicular magnet. **D)** Constant curvature deformation model. **E)** The gravitational force and base torque acting on the perpendicular magnet in axial view. The distributed body forces were not shown.

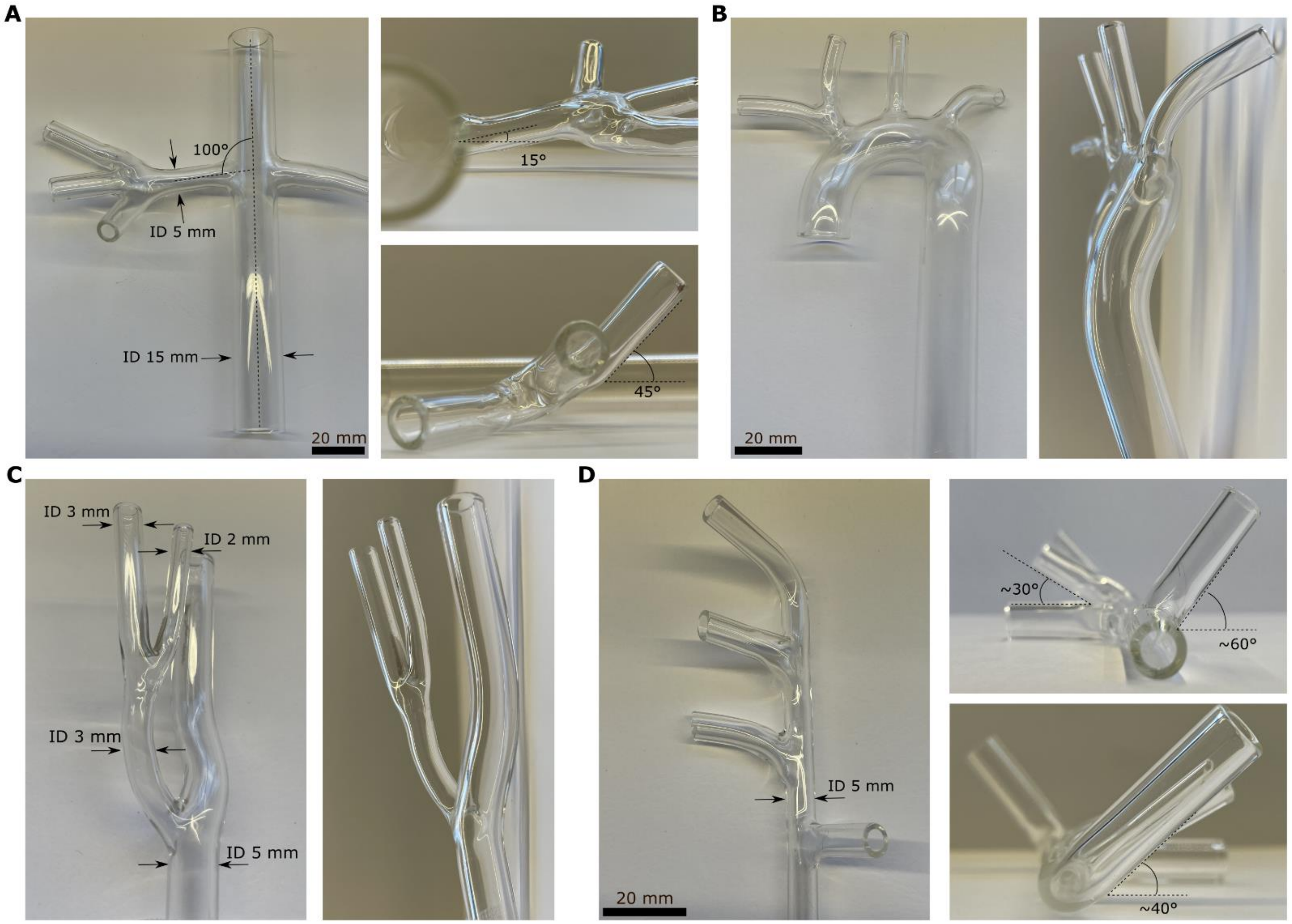

Figure S10. 3D glass vascular models. A) The renal artery model for the right kidney. The renal artery has been shown from top, front, and side views. **B)** Aortic arch model from top and side views. **C)** Common carotid artery model from top and size view. **D)** The middle cerebral artery model. The model is shown in the orientation it is used in the experiments. The branching angles are shown in the side views.

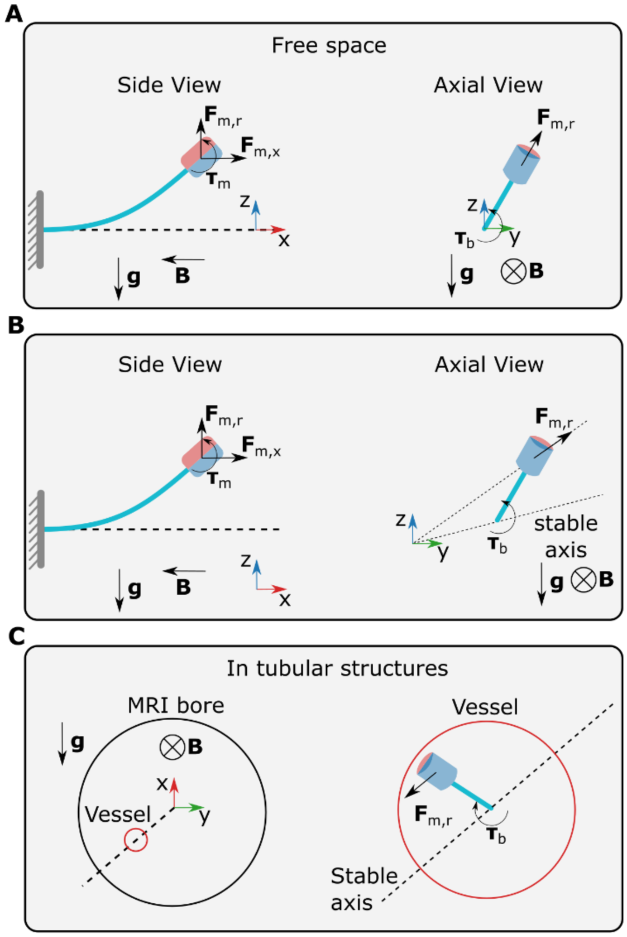

Figure S11. Effect of radial magnetic forces. A) Radial magnetic forces for the guidewire aligned with the MRI axis. The radial forces aligned with the fixed end of the guidewire. **B)** The radial magnetic forces for an off-centered guidewire. The stable axis connects the MRI axis and the guidewire's fixed end in the axial view. **C)** Radial magnetic forces on a guidewire in an off-centered vessel. The guidewire's fixed end is depicted as the center of the vessel; however, in reality, it could lean toward vessel walls in different directions depending on the vasculature network.

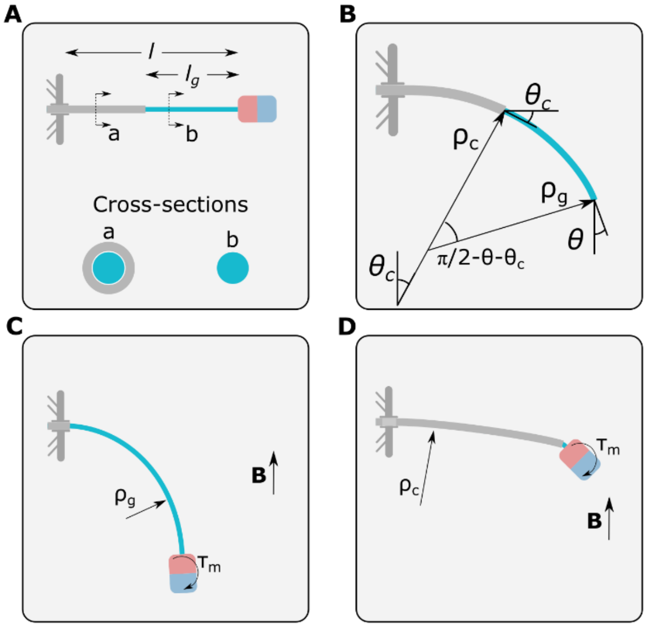

Figure S12. Free length control. A) The guidewire with supporting Teflon catheter. The fixed end of the guidewire is replaced with a fixed insertion point, where the catheter and guidewire can be inserted separately. The two cross-sections are shown: guidewire with catheter and only guidewire. **B)** The guidewire under a uniform magnetic field. Two segments of the guidewire have different constant curvatures due to the magnetic torque applied to the tip. **C)** The maximum guidewire deformation. The supporting catheter was pulled back, and the free length was only composed of the guidewire. **D)** The minimum guidewire deformation. The supporting catheter is inserted up to the tip magnet, and the free length is the composition of the guidewire and catheter together.

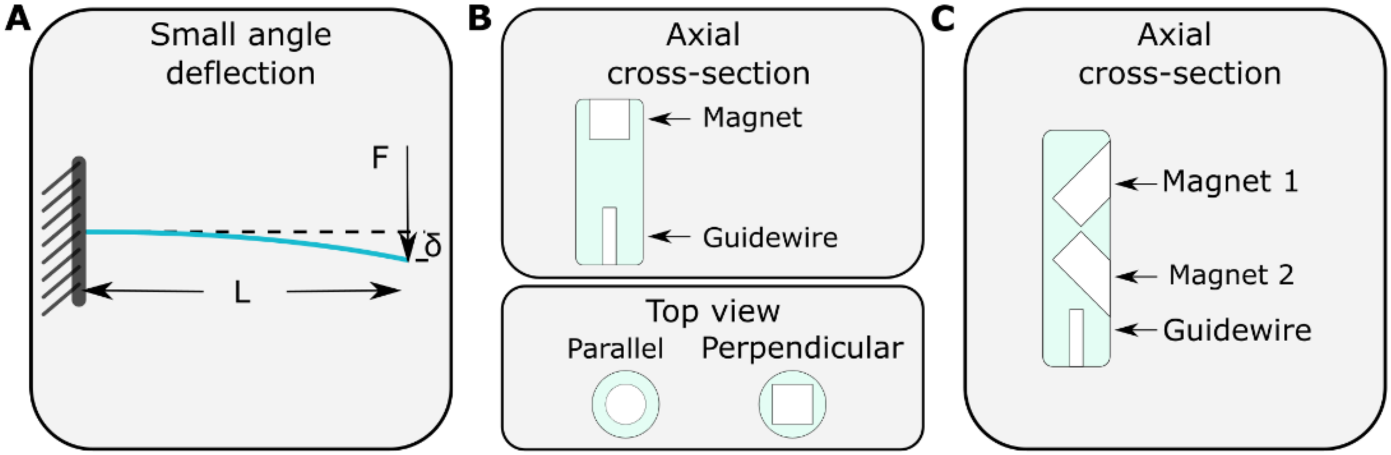

Figure S13. The guidewire and tip design. A) The small angle deflection model for stiffness characterization. B) The 3D printed tip magnet holder for single magnet guidewires. The axial cross-section showed the position of the magnet and the guidewire connection. The top view shows the shape of a cylindrical magnet cavity for parallel and perpendicular configurations. **C)** The 3D-printed tip magnet holder for double magnet guidewires. The magnets were aligned perpendicular to each other and 45° to the vertical axis of the magnet holder.

Supplementary Movie Captions

Movie S1. The guidewire with the parallel tip magnet was navigated to three target positions at a 7 T magnetic field by rotating the robotic platform. The third target was reached using assistive magnetic pulling force at the distal tip by moving the platform 2.5 T/m gradient line.

Movie S2. The guidewire with the parallel tip magnet was navigated to the first two target positions at a 0.5 T magnetic field, i.e., fringe field, by rotating the robotic platform. The third target could not be reached due to insufficient magnetic torque.

Movie S3. The guidewire with the perpendicular tip magnet was navigated to three target positions using magnetic bending and base twist. The field strength was used to control the guidewire bending by moving the platform in and out of the MRI scanner. The steering was achieved using a base twist.

Movie S4. The guidewire with the antiparallel tip magnet was navigated to three target positions using magnetic bending and base twist. Guidewire bending up to 160° has been shown at 7T, and steering was achieved with a base twist to different targets. Next, the magnet was remagnetized by physically constraining the tip during insertion into MRI. Then the guidewire was advanced to the last target. The magnetization direction of the tip was shown in the guidewire schematic.

Movie S5. The remagnetization process of a single parallel magnet guidewire was demonstrated in an s-shaped tube. The guidewire was advanced when the orientation was not locked with large magnetic torque when the platform was out of the MRI. Then the platform was moved into the MRI scanner to remagnetize the tip magnet. The remagnetization and orientation locking were observed during the guidewire insertion and the retraction. In addition, the magnet switched magnetization during insertion between antiparallel to parallel states. The magnetization direction of the tip was shown in the guidewire schematic.

Movie S6. The remagnetization process of a dual magnet guidewire was demonstrated in an s-shaped tube. The guidewire was advanced when the orientation was not locked with large magnetic torque when the platform was out of the MRI. Then the platform was moved into the MRI scanner to remagnetize the tip magnet. The remagnetization and orientation locking were observed during the guidewire insertion and the retraction. The magnet switches magnetization four times during insertion between parallel, perpendicular, and antiparallel states. The magnetization direction of the tip was shown in the guidewire schematic.

Movie S7. Steering into the renal artery using the guidewire with a perpendicular magnet was demonstrated under physiological flow. First, the guidewire was introduced in the descending aorta against the 100 ml/beat flow. Next, the guidewire was steered into the renal artery using a 1T magnetic field and base twist maneuver. Later, the magnetic gradient 15 T/m and 2 T magnetic field were used to bend the guidewire toward superior segmental arteries.

Movie S8. Steering into the renal artery using the guidewire with an antiparallel magnet was demonstrated under physiological flow. First, the guidewire was introduced in the descending aorta against the 100 ml/beat flow. Next, the guidewire was steered into the renal artery using a 0.5T magnetic field and base twist maneuver. Later, the 2 T magnetic field was used to bend the guidewire toward inferior segmental arteries.

Movie S9. The guidewire steering has been demonstrated in the aortic arch. The guidewire with a perpendicular magnet was inserted in the aortic arch against 100 ml/beat physiological flow. Then at 0.5 T magnetic field, the guidewire was

steering into LS and LCC arteries. Later the platform was moved to a 4 T magnetic field, and a guidewire was inserted into the RS artery. The guidewire could not be steered into the RCC due to orientation in the vessel.

Movie S10. The guidewire steering has been demonstrated in the LCC with 20 ml/beat physiological flow. The guidewire could be inserted directly into the ICA at 0.5 T. We moved the platform to 1 T and used a base twist to steer into ECA. Then guidewire could be steered directly to FA. Next, we moved the platform to a 2 T field to steer into OA and rotate the tip into the OA. Then the platform moved back to 0.5 T to ease insertion to a narrow 2 mm-diameter OA channel.

Movie S11. The guidewire steering has been demonstrated in the MCA with 20 ml/beat physiological flow. The guidewire with a perpendicular magnet was inserted through the internal carotid artery and steered into the M1 segment of the MCA using base twist at a 1T magnetic field. Later, guidewire pushed into the first M2 branch. However, the distal branches could not be reached due to the limited tip rotation.

Movie S12. The guidewire steering has been demonstrated in the MCA with 20 ml/beat physiological flow. The guidewire with an antiparallel magnet was inserted through the internal carotid artery. A supporting catheter was used for supporting the guidewire, and the guidewire was steered into the M1 segment of the MCA using base twist at 0.5 T magnetic field. With the above 90° tip rotation, the guidewire was inserted up to the last M2 branch. Then the tip magnet was constrained with the help of a supporting guidewire, and the platform was moved to 3 T to remagnetize the tip into parallel configuration. Then the guidewire pulled back and inserted the other M2 branches.

Movie S13. The guidewire insertion has been demonstrated in the renal cavity of an ex vivo porcine kidney during MR imaging. A guidewire with a perpendicular magnet was inserted through the urethra into the renal cavity. The guidewire reached the calyx in front of the renal cavity entrance due to the 90° tip orientation. The tip position was monitored using the image artifact caused by the tip magnet.

Movie S14. The guidewire steering has been demonstrated in the renal cavity of an ex vivo porcine kidney during MR imaging. First, a guidewire with an antiparallel magnet and supporting Teflon guidewire was inserted through the urethra into the renal cavity. Next, the guidewire reached the lower calyx, and the guidewire was steered to other calyces using free-length control. Then the guidewire was pulled back to the urethra and remagnetized into a parallel configuration without moving the kidney. Later, the guidewire was steered into the upper calyces in a parallel magnet configuration, and the free length control was used for steering different locations. Finally, the tip position was monitored using the image artifact caused by the tip magnet.

REFERENCES

1. M. E. M. K. Abdelaziz, L. Tian, M. Hamady, G. Z. Yang, B. Temelkuran, X-ray to MR: The progress of flexible instruments for endovascular navigation. *Prog. Biomed. Eng.* **3**, 032004 (2021).

2. M. Bock, F. K. Wacker, MR-guided intravascular interventions: Techniques and applications. *J. Magn. Reson. Imaging* **27**, 326–338 (2008).

3. L. Muller, M. Saeed, M. W. Wilson, S. W. Hetts, Remote control catheter navigation: Options for guidance under MRI. *J. Cardiovasc. Magn. Reson.* **14**, 33 (2012).

4. M. Weiger, K. P. Pruessmann, P. Boesiger, Cardiac real-time imaging using SENSE. *Magn. Reson. Med.* **43**, 177–184 (2000).

5. M. Bock, S. Volz, S. Zühlsdorff, R. Umathum, C. Fink, P. Hallscheidt, W. Semmler, MR-guided intravascular procedures: Real-time parameter control and automated slice positioning with active tracking coils. *J. Magn. Reson. Imaging* **19**, 580–589 (2004).

6. T. Heidt, S. Reiss, A. J. Krafft, A. C. Özen, T. Lottner, C. Hehrlein, R. Galmbacher, G. Kayser, I. Hilgendorf, P. Stachon, D. Wolf, A. Zirlik, K. Düring, M. Zehender, S. Meckel, D. von Elverfeldt, C. Bode, M. Bock, C. von zur Mühlen, Publisher correction: Real-time magnetic resonance imaging – guided coronary intervention in a porcine model. *Sci. Rep.* **9**, 18282 (2019).

7. H. C. Clogenson, J. J. van den Dobbelsteen, Catheters and guide wires for interventional MRI: Are we there yet? *Imaging Med.* **8**, 10.14303/IMAGING-MEDICINE.1000024 (2016).

8. Ö. Erin, M. Boyvat, M. E. Tiryaki, M. Phelan, M. Sitti, Magnetic resonance imaging system-driven medical robotics. *Adv. Intell. Syst.* **2**, 1900110 (2020).

9. J. Sheng, X. Wang, T.-M. L. Dickfeld, J. P. Desai, Towards the development of a steerable and MRI-compatible cardiac catheter for atrial fibrillation treatment. *IEEE Robot. Autom. Lett.* **3**, 4038–4045 (2018).

10. J. A. Bell, C. E. Saikus, K. Ratnayaka, V. Wu, M. Sonmez, A. Z. Faranesh, J. H. Colyer, R. J. Lederman, O. Kocaturk, A deflectable guiding catheter for real-time MRI-guided interventions. *J. Magn. Reson. Imaging* **35**, 908–915 (2012).

11. G. Fang, M. C. K. Chow, J. D. L. Ho, Z. He, K. Wang, T. C. Ng, J. K. H. Tsoi, P.-L. Chan, H.-C. Chang, D. T.-M. Chan, Y.-H. Liu, F. C. Holsinger, J. Y.-K. Chan, K.-W. Kwok, Soft robotic manipulator for intraoperative MRI-guided transoral laser microsurgery. *Sci. Robot.* **6**, eabg5575 (2021).

12. K. Ikuta, H. Ichikawa, K. Suzuki, T. Yamamoto, Safety active catheter with multi-segments driven by innovative hydro-pressure micro actuators. *Proc. IEEE Micro Electro Mech. Syst.* 10.1109/MEMSYS.2003.1189704 (2003).

13. P. E. Dupont, J. Lock, B. Itkowitz, E. Butler, Design and control of concentric-tube robots. *IEEE Trans. Robot.* **26**, 209–225 (2010).

14. H. Su, G. Li, D. C. Rucker, R. J. Webster, G. S. Fischer, A concentric tube continuum robot with piezoelectric actuation for MRI-guided closed-loop targeting. *Ann. Biomed. Eng.* **44**, 2863–2873 (2016).

15. M. Ho, A. B. McMillan, J. M. Simard, R. Gullapalli, J. P. Desai, Toward a meso-scale SMA-actuated MRI-compatible neurosurgical robot. *IEEE Trans. Robot.* **2011**, 1–10 (2012).

16. P. R. Buckley, G. H. McKinley, T. S. Wilson, W. Small IV, W. J. Benett, J. P. Bearinger, M. W. McElfresh, D. J. Maitland, Inductively heated shape memory polymer for the magnetic actuation of medical devices. *IEEE Trans. Biomed. Eng.* **53**, 2075–2083 (2006).

17. M. W. Wilson, A. B. Martin, P. Lillaney, A. D. Losey, E. J. Yee, A. Bernhardt, V. Malba, L. Evans, R. Sincic, M. Saeed, R. L. Arenson, S. W. Hetts, Magnetic catheter manipulation in the interventional MR imaging environment. *J. Vasc. Interv. Radiol.* **24**, 885–891 (2013).

18. M. F. Phelan III, M. E. Tiryaki, J. Lazovic, H. Gilbert, M. Sitti, Heat-mitigated design and Lorentz force-based steering of an MRI-driven microcatheter toward minimally invasive surgery. *Adv. Sci. (Weinh)* **9**, e2105352 (2022).

19. P. Moftakhar, P. Lillaney, A. D. Losey, D. L. Cooke, A. J. Martin, B. R. H. Thorne, R. L. Arenson, M. Saeed, M. W. Wilson, S. W. Hetts, New-generation laser-lithographed dual-axis magnetically assisted remote-controlled endovascular catheter for interventional MR imaging: In vitro multiplanar navigation at 1.5 T and 3 T versus X-ray fluoroscopy *Radiology* **277**, 842–852 (2015).

20. P. V. Lillaney, J. K. Yang, A. D. Losey, A. J. Martin, D. L. Cooke, B. R. H. Thorne, D. C. Barry, A. Chu, C. Stillson, L. Do, R. L. Arenson, M. Saeed, M. W. Wilson, S. W. Hetts, Endovascular MR-guided renal embolization by using a magnetically assisted remote-controlled catheter system. *Radiology* **281**, 219–228 (2016).

21. T. Liu, M. C. Çavuşoğlu, Three dimensional modeling of an MRI actuated steerable catheter system. *IEEE Int. Conf. Robot. Autom.* **2014**, 4393–4398 (2014).

22. T. Liu, R. Jackson, D. Franson, N. L. Poirot, R. K. Criss, N. Seiberlich, M. A. Griswold, M. C. Cavusoglu, Iterative Jacobian-based inverse kinematics and open-loop control of an MRI-guided magnetically actuated steerable catheter system. *IEEE/ASME Trans. Mechatronics.* **22**, 1765–1776 (2017).

23. V. Lalande, F. P. Gosselin, M. Vonthron, B. Conan, C. Tremblay, G. Beaudoin, G. Soulez, S. Martel, In vivo demonstration of magnetic guidewire steerability in a MRI system with additional gradient coils. *Med. Phys.* **42**, 969–976 (2015).

24. V. Lalande, F. P. Gosselin, S. Martel, Catheter steering using a magnetic resonance imaging system. *Annu. Int. Conf. IEEE Eng. Med. Biol.* **2010**, 1874–1877 (2010).

25. K. Zhang, A. J. Krafft, R. Umathum, F. Maier, W. Semmler, M. Bock, Real-time MR navigation and localization of an intravascular catheter with ferromagnetic components. *MAGMA* **23**, 153–163 (2010).

26. M. E. Tiryaki, M. Sitti, Magnetic resonance imaging-based tracking and navigation of submillimeter-scale wireless magnetic robots. *Adv. Intell. Syst.* **4**, 2100178 (2022).

27. A. Azizi, C. C. Tremblay, K. Gagné, S. Martel, Using the fringe field of a clinical MRI scanner enables robotic navigation of tethered instruments in deeper vascular regions. *Sci. Robot.* **4**, eaax7342 (2019).

28. J. J. Abbott, O. Ergeneman, M. P. Kummer, A. M. Hirt, B. J. Nelson, Modeling magnetic torque and force for controlled manipulation of soft-magnetic bodies. *IEEE Trans. Robot.* **23**, 1247–1252 (2007).

29. K. Samsami, S. A. Mirbagheri, F. Meshkati, H. C. Fu, Saturation and coercivity limit the velocity of rotating active magnetic microparticles. *Phys. Rev. Fluids.* **5**, 64202 (2020).

30. J. J. Abbott, E. Diller, A. J. Petruska, Magnetic methods in robotics. *Annu. Rev. Control. Robot. Auton. Syst.* **3**, 57–90 (2020).

31. B. D. Cullity, C. D. Graham, *Introduction to Magnetic Materials* (John Wiley & Sons, 2008).

32. Y. Kim, G. A. Parada, S. Liu, X. Zhao, Ferromagnetic soft continuum robots. *Sci. Robot.* **4**, eaax7329 (2019).

33. Y. Kim, E. Genevriere, P. Harker, J. Choe, M. Balicki, R. W. Regenhardt, J. E. Vranic, A. A. Dmytriw, A. B. Patel, X. Zhao, Telerobotic neurovascular interventions with magnetic manipulation. *Sci. Robot.* **7**, eabg9907 (2022).

34. G. Pittiglio, P. Lloyd, T. da Veiga, O. Onaizah, C. Pompili, J. H. Chandler, P. Valdastri, Patient-specific magnetic catheters for atraumatic autonomous endoscopy. *Soft Robot.* **9**, 1120–1133 (2022).

35. J. Lussi, M. Mattmann, S. Sevim, F. Grigis, C. De Marco, C. Chautems, S. Pané, J. Puigmartí-Luis, Q. Boehler, B. J. Nelson, A submillimeter continuous variable stiffness catheter for compliance control. *Adv. Sci. (Weinh)* **8**, e2101290 (2021).

36. S. Jeon, A. K. Hoshiar, K. Kim, S. Lee, E. Kim, S. Lee, J.-Y. Kim, B. J. Nelson, H.-J. Cha, B.-J. Yi, H. Choi, A magnetically controlled soft microrobot steering a guidewire in a three-dimensional phantom vascular network. *Soft Robot.* **6**, 54–68 (2019).

37. N. Kim, W. Lee, E. Jung, J. Kim, J. Park, G. Jang, Image-based feedback control of a magnetic catheter to enhance the path-following capability of the position and orientation at its distal part. *AIP Adv.* **9**, 125127 (2019).

38. J. Edelmann, A. J. Petruska, B. J. Nelson, Magnetic control of continuum devices. *Int. J. Rob. Res.* **36**, 68–85 (2017).

39. A. J. Petruska, J. J. Abbott, Optimal permanent-magnet geometries for dipole field approximation. *IEEE Trans. Magn.* **49**, 811–819 (2013).

40. E. Onrat, I. E. Uluışık, G. Ortug, The left vertebral artery arising directly from the aortic arch. *Transl. Res. Anat.* **24**, 100122 (2021).

41. M. E. Tiryaki, S. O. Demir, M. Sitti, Deep learning-based 3D magnetic microrobot tracking using 2D MR images. *IEEE Robot. Autom. Lett.* **7**, 6982–6989 (2022).

42. D. Folio, C. Dahmen, T. Wortmann, M. A. Zeeshan, K. Shou, S. Pane, B. J. Nelson, A. Ferreira, S. Fatikow, in *2011 IEEE/RSJ International Conference on Intelligent Robots and Systems* (2011), pp. 1297–1303.

43. O. Kraff, A. Fischer, A. M. Nagel, C. Mönninghoff, M. E. Ladd, MRI at 7 Tesla and above: Demonstrated and potential capabilities. *J. Magn. Reson. Imaging* **41**, 13–33 (2015).

44. M. J. Versluis, W. M. Teeuwisse, H. E. Kan, M. A. van Buchem, A. G. Webb, M. J. van Osch, Subject tolerance of 7 T MRI examinations. *J. Magn. Reson. Imaging* **38**, 722–725 (2013).

45. O. Al-Ahmad, M. Ourak, J. Van Roosbroeck, J. Vlekken, E. Vander Poorten, Improved FBG-based shape sensing methods for vascular catheterization treatment. *IEEE Robot. Autom. Lett.* **5**, 4687–4694 (2020).

46. J. Till, V. Aloi, C. Rucker, Real-time dynamics of soft and continuum robots based on Cosserat rod models. *Int. J. Rob. Res.* **38**, 723–746 (2019).

47. F. P. Beer, E. R. Johnston Jr, J. T. Dewolf, D. F. Mazurek, Mechanics of materials (2010).

48. D. Tong, A. Borum, M. K. Jawed, Automated stability testing of elastic rods with helical centerlines using a robotic system. *IEEE Robot. Autom. Lett.* **7**, 1126–1133 (2021).
